# Sodium-Glucose Cotransporter-2 Inhibitors and Arrhythmias: A Meta-Analysis of 38 Randomized Controlled Trials

**DOI:** 10.1016/j.jacadv.2025.101615

**Published:** 2025-02-22

**Authors:** Vikash Jaiswal, Song Peng Ang, Danisha Kumar, Novonil Deb, Akash Jaiswal, Amey Joshi, Yusra Minahil Nasir, Dhrubajyoti Bandyopadhyay, Erin D. Michos, Emelia J. Benjamin, Gregg C. Fonarow

**Affiliations:** a Department of Cardiovascular Research, Larkin Community Hospital, South Miami, Florida, USA; b Department of Internal Medicine, Rutgers Health/Community Medical Center, Toms River, New Jersey, USA; c Dow University of Health Sciences, Karachi, Pakistan; d North Bengal Medical College, West Bengal, India; e Department of Geriatric Medicine, All India Institute of Medical Science, New Delhi, India; f Michigan State University-Sparrow Hospital, Lansing, Michigan, USA; g Department of Internal Medicine, University of Oklahoma Health Science Center, Oklahoma City, Oklahoma, USA; h Massachusetts General Hospital, Harvard Medical School, Boston, Massachusetts, USA; i Division of Cardiology, Johns Hopkins University School of Medicine, Baltimore, Maryland, USA; j Department of Medicine, Cardiovascular Medicine, Boston Medical Center, Boston University Chobanian & Avedisian School of Medicine, Boston, Massachusetts, USA; k Epidemiology Department, Boston University School of Public Health, Boston, Massachusetts, USA; l Ahmanson-UCLA Cardiomyopathy Center, Ronald Reagan UCLA Medical Center, Los Angeles, California, USA

**Keywords:** atrial arrhythmia, heart failure, SGLT2i, sudden cardiac death, ventricular arrhythmia

## Abstract

**Background:**

Sodium-glucose cotransporter-2 inhibitors (SGLT2i) have shown promising results in reducing hospitalizations from heart failure (HF) and cardiovascular mortality. However, their effect on arrhythmia and sudden cardiac death (SCD) is not well established.

**Objectives:**

The authors sought to evaluate the association between SGLT2i and the risk of arrhythmias and SCD in patients with type 2 diabetes mellitus, HF, or chronic kidney disease.

**Methods:**

We performed a systematic literature search on PubMed, EMBASE, and Scopus for relevant randomized controlled trials from inception until February 10, 2023. ORs and 95% CIs were pooled using a random effect model.

**Results:**

A total of 38 randomized controlled trials with 88,704 patients (48,435 in the SGLT2i group and 40,269 in the control group) were included in the study. The mean age of patients among SGLT2i and control groups was 56.8 and 56.7 years, respectively. The mean follow-up duration was 1.6 years. Pooled analysis of primary and secondary outcomes showed that SGLT2i significantly reduced the risk of incident atrial arrhythmia (OR: 0.85 [95% CI: 0.75-0.98], *P* = 0.02), SCD (OR: 0.72 [95% CI: 0.55-0.94], *P* = 0.02) compared with placebo. However, the risk of ventricular arrhythmia (OR: 1.03 [95% CI: 0.84-1.26], *P* = 0.77) and cardiac arrest (OR: 0.94 [95% CI: 0.72-1.23] *P* = 0.67) was comparable between both groups of patients.

**Conclusions:**

SGLT2i therapy was associated with an overall lower risk of atrial arrythmia and SCD in patients with type 2 diabetes mellitus and/or HF or chronic kidney disease. However, SGLT2i therapy was not associated with a lower risk of ventricular arrhythmia.

Cardiovascular events, including atrial and ventricular arrhythmias and sudden cardiac death (SCD), are more common in those affected by diabetes mellitus (DM).^1^^,^^2^ Long-term effects of type 2 DM (T2DM) can also contribute to heart failure (HF) and coronary artery disease that predispose to SCD.^3^ Cardiovascular risk factor control was found to contribute significantly to decreasing mortality rates in men and women with DM.^1^ Despite these interventions, SCD was reported to be twice as common in persons with DM and nearly 6 to 9 times more common in persons with HF compared to the general population. The leading cause of SCD in these patients was noted to be related to ventricular arrhythmias (VAs).^4^^,^^5^ Chronic kidney disease (CKD) is also a risk factor for SCD with the rate of death increasing proportionately to the stage of CKD. A recent report also suggested that in hemodialysis units, SCD occurs in 7 per 100,000 hemodialysis sessions. Sodium-glucose cotransporter-2 inhibitors (SGLT2i) have recently been approved for their use in slowing the progression of CKD and also reducing cardiovascular mortality in patients with CKD.^6^

Recent trials studying the effect of SGLT2i in patients with T2DM, HF, and/or CKD have shown promising results in decreasing HF hospitalizations, cardiovascular death, and all-cause mortality.^7^, ^8^, ^9^, ^10^, ^11^, ^12^, ^13^, ^14^ Clinical and experimental studies have suggested this to be largely attributed to SGLT2i’s properties of modulating ventricular remodeling, decreasing sympathetic activity, decreasing oxidative stress and inflammation, and improving renal function.^15^ With new evidence from the recent study of EMPA Kidney (Heart and Kidney Protection with Empagliflozin, 2023), EMPEROR-Reduced (Empagliflozin Outcome Trial in Patients with Chronic Heart Failure and a Reduced Ejection Fraction, 2020), DAPA-CKD (Dapagliflozin and Prevention of Adverse Outcomes in Chronic Kidney Disease, 2020), DELIVER (Dapagliflozin Evaluation to Improve the Lives of Patients with Preserved Ejection Fraction Heart Failure, 2022), and EMPEROR-Preserved (Empagliflozin Outcome Trial in Patients with Chronic Heart Failure with Preserved Ejection Fraction, 2021), we aimed to re-evaluate the effect of SGLT2i in preventing SCD and arrhythmias in patients with T2DM, HF, and/or CKD.^11^^,^^16^, ^17^, ^18^, ^19^ We therefore conducted a meta-analysis of randomized controlled trials (RCTs) from inception to date to examine the association between SGLTi’s treatment and the incidence of SCD and arrhythmias.

## Methods

This meta-analysis was conducted and reported following the PRISMA (Preferred Reporting Items for Systematic Review and Meta-Analysis) 2020 guidelines and performed according to established methods, as described previously.^20^, ^21^, ^22^, ^23^ The prespecified study protocol was registered in the PROSPERO (CRD42023437142).

### Search strategy

We conducted a systematic literature search in PubMed, Embase, and ClinicalTrial.gov using predefined MESH terms by using “AND” and “OR.” The following search terms were used: “Sodium-Glucose Cotransporter-2 Inhibitor” OR “SGLT2i” AND “Heart failure” OR “HFrEF” OR “HFpEF” AND “Diabetes Mellitus" OR “T2DM” AND “Chronic Kidney Disease” OR “CKD” AND “Sudden Cardiac Death” AND “Arrhythmias” OR “Atrial Fibrillation” OR “Atrial Flutter” OR Ventricular Fibrillation” OR “Ventricular Fibrillation” OR “Ventricular Arrhythmia.” We queried databases from their search inception up until February 10, 2023, without any restrictions on the language of the studies. Search strategies are listed in Supplemental Table 1.

All the studies were carefully screened and exported to the Mendeley reference manager used to handle searched citations. A manual check was carried through to cross-check for any remaining duplicates. Two reviewers (V.J. and N.D.) reviewed the papers based on the title and abstract. Discrepancies regarding the inclusion of studies were arbitrated by another author (A.J.).

### Eligibility criteria

We included studies with adult patients ≥18 years of age. All RCTs were eligible if they included patients with T2DM and/or HF or CKD. It was decided to include studies with 2 arms in one with SGLT2i as an intervention, and placebo or control as a comparator. Studies must have reported outcomes of interest (primary and secondary outcomes). Selected studies compared patients with varying baseline characteristics and pathologies.

Studies that were performed on animals, or reviews, case reports, case series, studies on patients <18 years, studies with a single arm or without SGLT2i as an intervention, studies with sotagliflozin (a dual sodium-glucose cotransporter-2 and 1 inhibitor) as an intervention, and studies without outcomes of interest were excluded from the review.

### Clinical outcomes

The primary outcome of this meta-analysis was atrial arrhythmia (which includes atrial fibrillation and flutter). Secondary outcomes included VA (which includes ventricular fibrillation, ventricular flutter, and ventricular tachycardia), SCD, and cardiac arrest.

### Data extraction and quality assessment

Two authors (N.D. and V.J.) extracted the following data: study type, author, study location, study follow-up duration, patient characteristics (number, age, sex, and comorbidities), and primary and secondary outcomes. Two investigators (D.H. and V.J.) independently appraised the potential risk of bias using version 2 of the Cochrane risk of bias tool for RCTs.^24^ We then classified studies as low, moderate, or high quality after the evaluation.

### Statistical analysis

Baseline continuous variables were summarized in mean ± SD, whereas dichotomous variables were described in frequency or percentage. We performed a conventional meta-analysis for primary and secondary outcomes and adopted the DerSimonian and Laird random effect model for the study variations.^25^ For studies with zero-events in either treatment of control groups, a continuity correction (0.5) was applied to each cells. Outcomes were reported as pooled OR, and their corresponding 95% CI. Statistical significance was met if the 95% CI did not cross the numeric “1” and the 2-tailed *P* value was <0.05. We considered a 2-tailed *P* value of <0.05 to be statistically significant. The heterogeneity among studies was assessed by Higgins's I-squared (I^2^) statistical model with I^2^ values. As a guide, I^2^ <25% indicated low, 25% to 50% moderate, and >50% high heterogeneity.^26^ Further sensitivity analyses were performed using a leave-one-out method to check the robustness of the results. Subgroup analyses were implemented to explore the causes of heterogeneity of primary and secondary outcomes based on type of SGLT2i and follow-up period. Assessment of publication bias was via visualization of the funnel plot.^27^ All statistical work, inclusive analysis, and graphical illustrations were conducted using STATA (version 17.0, StataCorp).^28^

## Results

### Baseline characteristics of patients in included studies

**Figure 1:**
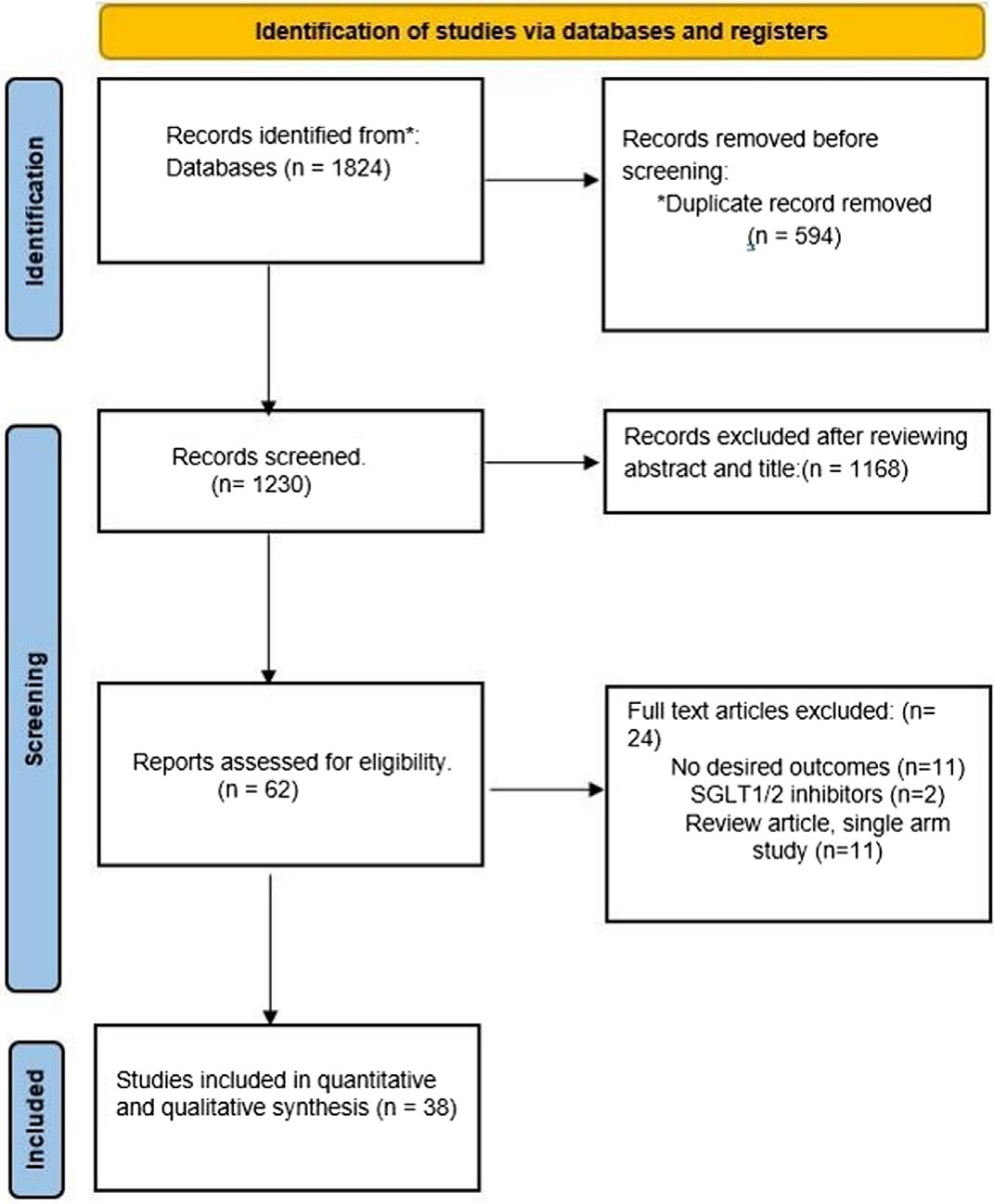
Preferred Reporting Items for Systematic Review and Meta-Analysis Flow Diagram of Search Among Different Databases

The initial search strategy yielded 1,824 articles, of which 594 duplicates were removed, and 1,168 articles were excluded after the title and abstract screening. The full-text review was performed on the remaining 62 studies, after which 24 studies were excluded from the final review and analysis for the following reasons: lack of appropriate comparison arm, review articles, lack of follow-up data, or lack of outcome of interest (Figure 1).

In summary, 38 studies met the eligibility criteria and were included in the final analysis. All of them were RCTs, of which dapagliflozin was used as an intervention in 13 studies,^7^^,^^9^^,^^16^^,^^17^^,^^29^, ^30^, ^31^, ^32^, ^33^, ^34^, ^35^, ^36^, ^37^ canagliflozin in 10 studies,^8^^,^^38^, ^39^, ^40^, ^41^, ^42^, ^43^, ^44^, ^45^ empagliflozin in 11 studies,^11^^,^^18^^,^^19^^,^^46^, ^47^, ^48^, ^49^, ^50^, ^51^, ^52^, ^53^ and ertugliflozin in 4 studies.^54^, ^55^, ^56^, ^57^ The total number of patients was 88,704, with 48,435 patients in the SGLT2i group and 40,269 in the control group. The mean age of patients among SGLT2i and control groups was 56.8 and 56.7 years, and the number of males in SGLT2i and control groups was 58.8% and 41.2%, respectively. The mean follow-up duration was 1.6 years. The study characteristics, patients’ demographics, and comorbidities are presented in Table 1. The quality assessment using Cochrane risk of bias showed that there was a low risk of bias across all included studies (Supplemental Figure 1).

**Table 1:** Baseline Demographics, Comorbidities, and Study Characteristics of Studies Included in the Meta-Analysis

Study/First Author, Year	Drug Used	Dose (mg)	Sample Size	Total Sample Size	Age, y	Male	DM	CKD	HF	Follow-Up (y)
DELIVER, 2022	Dapagliflozin	10	3,131	6,263	71.8	1,767	1,401	-	-	2.3
	Placebo		3,132		71.5	1,749	1,405	-	-	
EMPEROR Preserved	Empagliflozin	10	2,991	5,988	71.8	1,659	1,466	-	2,996	2.18
	Placebo		2,997		71.9	1,653	1,472	-	2,991	
EMPA-KIDNEY, 2023	Empagliflozin	10	3,304	6,609	63.4	2,207	-	-	-	2
	Placebo		3,305		63.3	2,210	-	-	-	
EMPA-TROPISM, 2021	Empagliflozin	10	40	80	64.2	27	-	-	40	0.5
	Placebo		40		59.9	27	-	-	40	
EMPEROR-REDUCED	Empagliflozin	10	1,863	3,726	66.5	1,411	-	-	-	3
	Placebo		1,863		66.5	1,426	-	-	-	
Bailey, 2013	Dapagliflozin	2.5/5/10	409	546	53.9	216	-	-	-	2
	Placebo		137		53.7	76	-	-	-	
CANTATA-D, 2013	Canagliflozin	100/300	735	1,101	55.4	339	-	-	-	0.5
	Placebo		366		55.5	172	-	-	-	
CANTATA-MSU, 2013	Canagliflozin	100/300	313	469	56.7	239	-	-	-	1
	Placebo		156		56.8	76	-	-	-	
CANTATA-SU, 2013	Canagliflozin	50/100/200/300	968	1,420	56.2	493	-	-	-	0.2
	Placebo		452		56.3	263	-	-	-	
CANVAS, 2017	Canagliflozin	100/300	2,888	4,329	63.2	3,759	-	-	-	5.7
	Placebo		1,441		62.3	956	-	-	-	
CANVAS-R, 2017	Canagliflozin	100/300	2,904	5,807	63.9	1,854	-	-	-	2
	Placebo		2,903		64	1,794				
Cefalu, 2015	Dapagliflozin	10	460	922	62.8	316	-	-	-	1
	Placebo		462		63	316	-	-	-	
CREDENCE, 2019	Canagliflozin	100	2,200	4,397	63	2,907	-	-	-	2.6
	Placebo		2,197		63.2	1,467	-	-	-	
DAPA-CKD, 2020	Dapagliflozin	10	2,149	4,298	61.8	1,443	1,455	-	235	2.4
	Placebo		2,149		61.9	1,436	1,451	-	233	
DAPA-HF, 2019	Dapagliflozin	10	2,368	4,736	66.2	1,804	993	962	1,124	1.5
	Placebo		2,368		66.5	1,823	990	964	1,127	
DECLARE-TIMI 58, 2018	Dapagliflozin	10	8,574	17,143	63.9	3,591	-	-	852	4.2
	Placebo		8,569		64	3,596	-	-	872	
CANTATA D2, 2013	Canagliflozin	300	377	755	56.5	207	-	-	-	2
	Placebo		378		56.6	215	-	-	-	
DURATION-8, 2016	Dapagliflozin	10	231	461	54.15	110	-	-	-	0.5
	Placebo		230		54	116	-	-	-	
EMPA-HEART CardioLink6, 2019	Empagliflozin	10	49	97	64	44	-	0	2	0.5
	Placebo		48		64	46	-	2	4	
EMPA-REG, 2015	Empagliflozin	10/25	4,687	7,020	63.1	5,016	-	607	-	3.1
	Placebo		2,333		63.2	1,680	-	605	-	
EMPA-REG BASALTM, 2015	Empagliflozin	10/25	324	494	59.25	186	-	-	-	1.5
	Placebo		170		58.1	80	-	-	-	
EMPA-REG H2H-SU, 2018	Empagliflozin	25	767	1,547	56.2	432	-	-	-	4
	Placebo		780		55.7	421	-	-	-	
EMPA-REG PIO, 2015	Empagliflozin	10/25	333	498	54.5	168	-	-	-	0.5
	Placebo		165		54.6	73	-	-	-	
EMPA-REG RENAL, 2014	Empagliflozin	10/25	419	738	62.6	249	-	-	-	1
	Placebo		319		64.1	181	-	-	-	
Ferrannini, 2010	Dapagliflozin	2.5/5/10	410	485	52.6	198	-	-	-	2
	Placebo		75		52.7	31	-	-	-	
Januzzi, 2017	Canagliflozin	100/300	450	666	64	248	-	-	-	2
	Placebo		216		63.2	133	-	-	-	
Leiter, 2014	Dapagliflozin	10	482	965	63.8	321	-	-	86	0.5
	Placebo		483		63.6	323	-	-	66	
Mathieu, 2015	Dapagliflozin	10	160	320	55.1	146	-	-	-	1
	Placebo		160		55	76	-	-	-	
Muller-Wieland, 2018	Dapagliflozin	10	312	625	57.4	202	-	-	-	1
	Placebo		313		58.6	206	-	-	-	
Nauck, 2013	Dapagliflozin	2.5/5/10	406	814	58	221	-	-	-	1
	Placebo		408		59	220	-	-	-	
Rosenstock, 2012	Canagliflozin	50/100/200/300	321	386	53.08	167	-	-	-	1
	Placebo		65		53.3	31	-	-	-	
Rosenstock, 2016	Canagliflozin	100/300	475	712	54.7	230	-	-	-	0.5
	Placebo		237		55.2	116	-	-	-	
Softeland, 2017	Empagliflozin	10/25	222	332	55.2	137	332	166	-	0.5
	Placebo		110		55.9	60				
VERTIS FACTORIAL, 2018	Ertugliflozin	5/15	498	745	55.2	261	-	-	-	1
	Placebo		247		54.8	153	-	-	-	
VERTIS MET, 2018	Ertugliflozin	5/15	412	621	56.7	190	-	-	-	2
	Placebo		209		56.5	98	-	-	-	
VERTIS RENAL, 2018	Ertugliflozin	5/15	313	467	67.3	231	-	-	-	1
	Placebo		154		67.5	72	-	-	-	
Wilding, 2012	Dapagliflozin	2.5/5	610	807	59.3	287	-	-	-	0.5
	Placebo		197		58.5	98	-	-	-	
VERTIS SU, 2018	Ertugliflozin	5/15	880	1,315	58.5	470	-	-	-	0.5
	Placebo		435		57.8	213	-	-	-	

CKD = chronic kidney disease; DM = diabetes mellitus; HF = heart failure.

### Meta-analysis on primary and secondary outcomes

**Figure 2:**
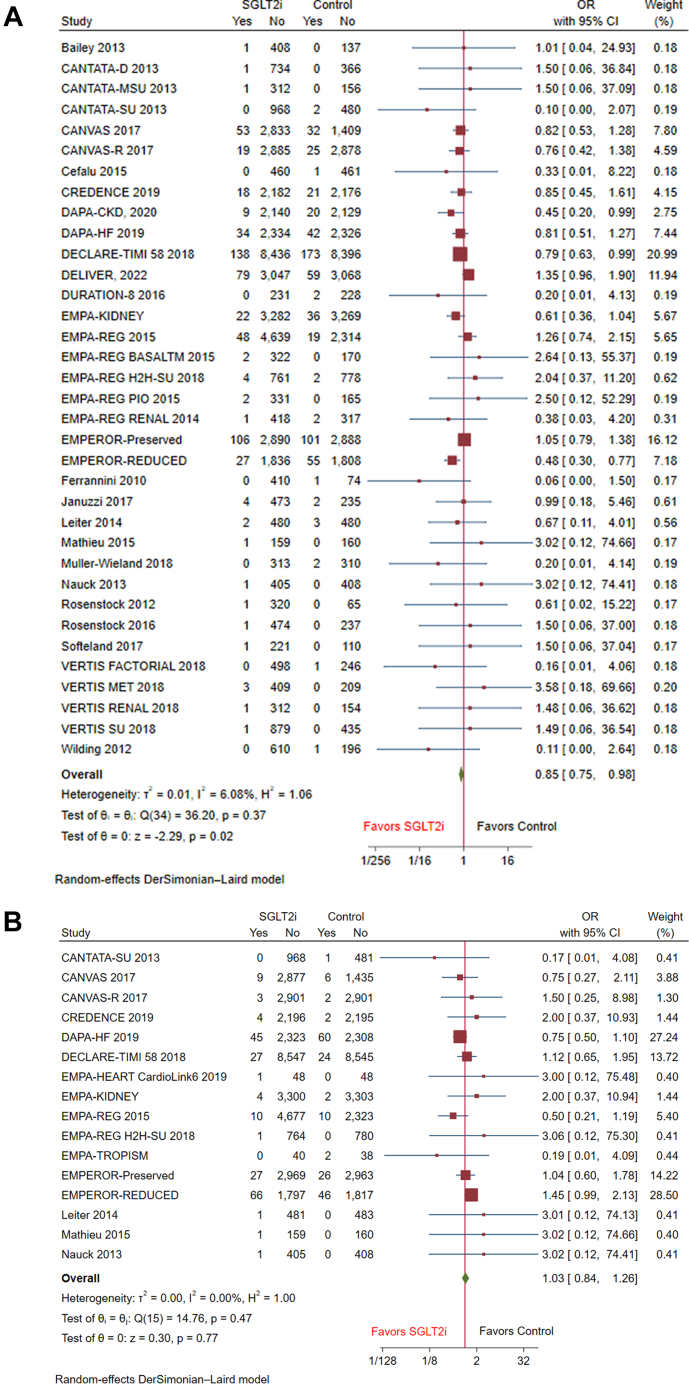
Forest Plots of Primary Outcomes Including Atrial and Ventricular Arrhythmias Forest plots of primary outcomes including (A) atrial arrhythmia, (B) ventricular arrhythmia. SGLT2i = sodium-glucose cotransporter-2 inhibitors.

Among the primary outcomes, pooled analysis of 38 trials showed that SGLT2i was associated with a significant reduction in the incidence of atrial arrhythmia (OR: 0.85 [95% CI: 0.75-0.98], *P* = 0.02, I^2^ = 6.08%) compared with the control group with minimal heterogeneity across studies (Figure 2A). Nevertheless, there was no significant association between SGLT2i and the incidence of VAs (OR: 1.03 [95% CI: 0.84-1.26], *P* = 0.77, I^2^ = 0) with minimal heterogeneity observed (Figure 2B).

**Figure 3:**
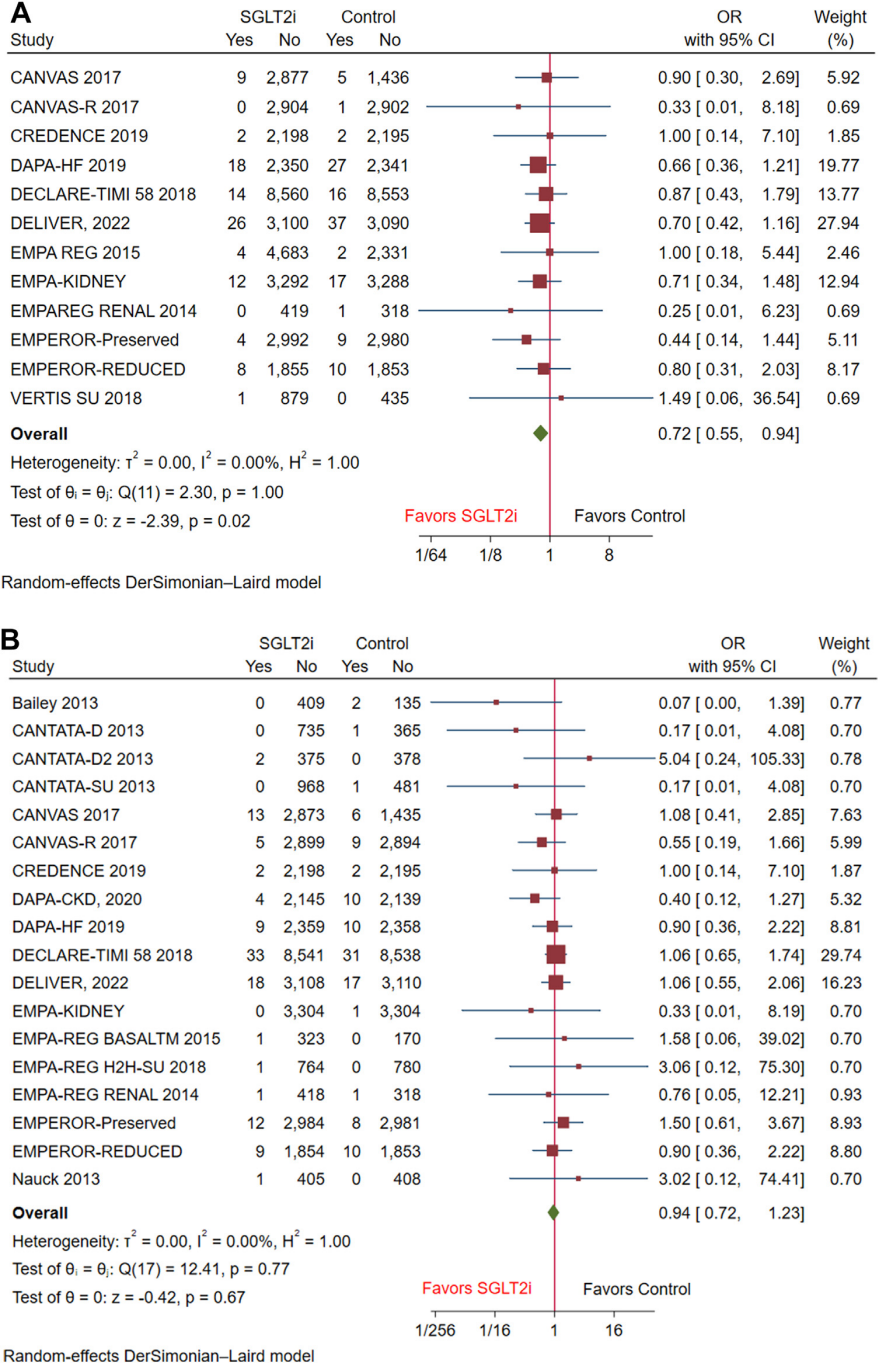
Forest Plots of Secondary Outcomes Including Sudden Cardiac Death and Cardiac Arrest Forest plots of secondary outcomes including (A) sudden cardiac death, (B) cardiac arrest. Abbreviation as in Figure 2.

In terms of secondary outcomes, SGLT2i was associated with a significant reduction in the incidence of SCD (OR: 0.72 [95% CI: 0.55-0.94], *P* = 0.02, I^2^ = 0) compared to the control group (Figure 3A). However, there was no significant association between SGLT2i and the incidence of cardiac arrest (OR: 0.94 [95% CI: 0.72-1.23], *P* = 0.67, I^2^ = 0) (Figure 3B).

### Meta-analysis based on components of atrial arrhythmias and ventricular arrhythmias

The pooled analysis shows that SGLT2i significantly reduced the incidence of atrial fibrillation (OR: 0.87 [95% CI: 0.76-0.98], *P* = 0.03, I^2^ = 0%), but not atrial flutter (OR: 0.83 [95% CI: 0.60-1.15], *P* = 0.27, I^2^ = 6.60%) compared with the control group (Supplemental Figures 2A and 2B). However, the risk of incidence of ventricular fibrillation (OR: 1.12 [95% CI: 0.73-1.70], *P* = 0.60, I^2^ = 0%), ventricular flutter (OR: 0.83 [95% CI: 0.20-3.39], *P* = 0.80), I^2^ = 0%), and ventricular tachycardia (OR: 1.00 [95% CI: 0.77-1.29], *P* = 0.98, I^2^ = 8.76%) was comparable between both the groups of patients (Supplementary Figures 3A to 3C).

### Subgroup analysis

Subgroup analyses were performed on primary and secondary outcomes based on the type of SGLT2i used. For atrial arrhythmias, there were no significant differences (*P* = 0.98) based on the type of SGLT2i used including canagliflozin (OR: 0.81 [95% CI: 0.60-1.09]), dapagliflozin (OR: 0.81 [95% CI: 0.61-1.11]), empagliflozin (OR: 0.85 [95% CI: 0.60-1.21]), and ertugliflozin (OR: 1.12 [95% CI: 0.23-5.39]) (Supplemental Figure 4).

In addition, there were no significant differences observed for the incidence of VA based on the types of SGLT2i including canagliflozin (OR: 0.95 [95% CI: 0.44-2.06]), dapagliflozin (OR: 0.85 [95% CI: 0.69-1.21]), and empagliflozin (OR: 1.11 [95% CI: 0.75-1.64]) with no significant subgroup difference (*P* = 0.68) (Supplemental Figure 5). After stratification by type of SGLT2i used, there were no significant differences in the incidence of SCD and cardiac arrest (Supplemental Figures 6 and 7).

We stratified the studies into subgroups of follow-up period of less than or equal to 1 year and more than 1 year, respectively. Overall, there was no significant difference between these subgroups based on follow-up period on the primary and secondary outcomes including atrial arrhythmia, cardiac arrest, SCD, sudden death, and ventricular fibrillation (Supplemental Figures 8 to 11).

### Sensitivity analyses and publication bias

Sensitivity analyses were carried out to test the robustness of the primary analysis. After leave-one-out analysis, the results of meta-analysis on the incidence of atrial arrhythmia remained largely unchanged and statistically significant, except when excluding DECLARE-TIMI 2018 and EMPEROR-REDUCED, where results became marginally insignificant (Supplemental Figure 12). Similarly for SCD, results were marginally insignificant after removal of study by DELIVER 2022 (Supplemental Figure 13). Otherwise, results of cardiac arrest and VA remained consistent with primary analysis, confirming the robustness of statistical analysis (Supplemental Figures 14 and 15).

A separate analysis has been performed by removing zero events from the primary and secondary outcomes. It was found that risk of atrial arrhythmia became nonsignificant, although showing a trend of nonsignificant reduction among SGLT2i. However, the risk of SCD, cardiac arrest, and VA remained consistent with primary analysis (Supplemental Figures 16 to 19).

Assessment of publication bias through funnel plot visualization showed that there was no evidence of publication bias, with minimal or no funnel plot asymmetry observed for all the outcomes (Supplemental Figures 20 to 23).

## Discussion

The present meta-analysis of 38 RCTs involving 88,704 patients shows that SGLT2i significantly lowered the risk of incident atrial arrhythmia and SCD (Central Illustration). Specifically, we observed that SGLT2i reduced the risk of atrial fibrillation. Additionally, no significant association was found between SGLT2i and the incidence of VAs, and cardiac arrest. The risk of ventricular fibrillation, ventricular flutter, and ventricular tachycardia was comparable between both groups of patients. These findings lend further evidence that SGLT2i therapy can provide clinically relevant risk reductions in atrial arrhythmias and SCD.

**Central Illustration:**
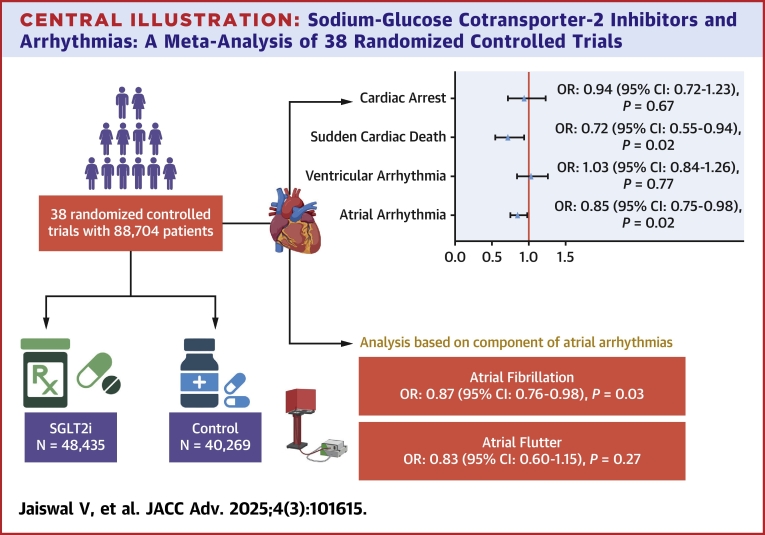
Sodium-Glucose Cotransporter-2 Inhibitors and Arrhythmias: A Meta-Analysis of 38 Randomized Controlled Trials Abbreviation as in Figure 2.

A meta-analysis by Sfairopoulos et al^58^ in 2021 suggested that SGLT2i therapy was not associated with a lower risk of SCD or VAs in patients with T2DM and/or HF and/or CKD. Since the previous meta-analysis in 2021, new data became available from landmark RCTs published such as DELIVER, EMPEROR PRESERVED, DAPA-CKD, and EMPEROR-REDUCED. Our meta-analysis incorporates a substantial body of new evidence derived from these rigorously conducted RCTs. In contrast, our study reported that SGLT2i therapy was associated with a statistically significant reduction in the incidence of SCD in patients with T2DM and/or HF. Another meta-analysis by Oates et al,^59^ 2023 reported that their analysis did not demonstrate a reduction in the incidence of arrhythmic events (including both atrial arrhythmias and VAs) in patients receiving SGLT2i therapy. Our study shows a significant reduction in the incidence of atrial arrhythmias in patients receiving SGLT2i therapy. Our primary research findings, after including DAPA-CKD,^16^ DELIVER,^17^ and EMPEROR-Preserved^18^ trials, are consistent with the meta-analysis by Fernandes et al,^60^ as both report a lower incidence of atrial arrhythmias and SCD when treated with SGLT2i, indicating the robustness of our results. Health care professionals and policymakers may be more inclined to consider the findings from the study when making decisions about the use of SGLT2i in patients with T2DM, HF, and/or CKD. It also provides additional insights into the effectiveness of different types of SGLT2i in patient populations with T2DM or HF.

These favorable effects of SGLT2i on atrial arrhythmias and SCD may be ascribed to the SGLT2i's capacity to impact ventricular remodeling, diminish sympathetic activity, mitigate oxidative stress and inflammation, and enhance renal function.^15^ Increased susceptibility to arrhythmias is commonly exacerbated by cardiac remodeling, hypertrophy, and dysfunction caused by fibrosis. SGLT2i offer indirect protection against arrhythmias by mitigating the risk of atrial fibrillation and preserving myocardial function.^61^ SGLT2i have been found to exert a positive impact on the activity of phospholamban, leading to the phosphorylation of calcium/calmodulin-dependent protein kinase II and consequent modulation of sarcoplasmic calcium release. As a result, these inhibitors effectively mitigate calcium overload, thereby reducing the incidence of arrhythmias.^62^ Additionally, SGLT2i are potent late I_*Na*_ inhibitors and are highly selective for late I_*Na*_, signifying that SGLT2i could markedly decrease the risk of arrhythmias.^63^

SGLT2i have been widely acknowledged for their cardioprotective properties, which can be attributed to a range of interconnected pathways that may collectively reduce the risk of SCD.^64^ These pathways involve multiple mechanisms: firstly, the induction of hemodynamic effects leading to plasma volume contraction and subsequent blood pressure reduction, which effectively lessens both preload and afterload on the heart.^65^, ^66^, ^67^ Secondly, the inhibition of sodium-hydrogen exchange within myocardial cells has been associated with mitigating myocardial hypertrophy, fibrosis, adverse remodeling, systolic dysfunction, and HF.^65^, ^66^, ^67^ Lastly, there is the potential for suppressing the sympathetic nervous system, adding to the overall cardioprotective impact of SGLT2i.^67^^,^^68^ Altogether, these intricate mechanisms underscore the considerable cardioprotective benefits of SGLT2i, offering a promising avenue for decreasing the incidence of SCD.

Our study showed that the use of SGLT2i did not result in a significant difference in the incidence of VAs. This may be due to a small number of studies; hence, reflecting a lack of power rather than a lack of effect. It has been established that left ventricular volumes, both end-diastolic and end-systolic, serve as surrogate indicators for unfavorable ventricular remodeling in HF, showing a strong correlation with the efficacy of specific drug or device therapies on patient survival.^69^ Furthermore, RCTs on mortality suggest a higher likelihood of neutral or favorable effects with increasing mean left ventricular ejection fraction.^70^ As a result, it is reasonable to speculate that the impact of SGLT2i therapy on left ventricular end-diastolic volume and left ventricular ejection fraction may not be substantial. Consequently, its potential as an anti-arrhythmic agent for VAs might be relatively modest overall. Nevertheless, further rigorously designed clinical studies with ample statistical power and extended follow-up periods are imperative to draw definitive conclusions regarding the potential reduction of VA incidence by SGLT2is.

The generalizability of our findings is strengthened by the inclusion of data from 38 RCTs, encompassing a diverse population of men and women with diabetes, CKD, or HF. While males comprised a larger proportion of participants across most trials, a substantial number of females were also included, ensuring the applicability of results to both sexes. However, younger individuals, older adults over 80 years, and patients from low-resource settings remain underrepresented, which may limit generalizability to these groups. The consistent dosing regimens and representation of various SGLT2 inhibitors enhance applicability across clinical contexts, but variability in follow-up durations and limited data on patients with complex comorbidities suggest caution when extrapolating to populations with chronic or multifaceted medical conditions. Further research should aim to include underrepresented groups and focus on long-term outcomes to ensure broader applicability of the findings.

### Strength

This meta-analysis, encompassing 38 RCTs, offers a robust and comprehensive evaluation of the relationship between SGLT2i and the incidence of arrhythmias, providing a depth of insight beyond that of individual studies. The implementation of rigorous methodology and analysis including sensitivity, subgroup analyses, and evaluation of potential publication bias ensured a systematic assessment of available data and thus enhancing the reliability of our findings.

### Study Limitations

Nevertheless, several limitations should be kept in mind upon interpretations of our findings. The included RCTs have varying study design, patient population, and endpoints. Therefore, the pooled results may be influenced by this inherent heterogeneity. Furthermore, with a median follow-up period of 1 year, it may not be sufficient to capture potentially delayed effects of SGLT2i, thus may underestimate the benefits of SGLT2i. Furthermore, these RCTs were designed with adequate power to detect differences in their primary endpoints, which means secondary or tertiary endpoints, including the ones of our interest, might not have been sufficiently powered. While meta-analysis can enhance the statistical power by pooling data across studies, the absence of specific outcomes of interest in individual studies still limits the overall power for particular endpoints such as SCD and cardiac arrest. Lastly, the low-event rate observed in certain endpoints may introduce challenge in deriving a reasonable inference. Due to the presence of studies with zero events, we applied continuity correction to facilitate the analysis. It is worth noting that such method allowed the inclusion of more studies but may introduce bias, thus may underestimate or overestimate the true effect size.

## Conclusions

SGLT2i therapy was associated with an overall lower risk of atrial arrhythmia and SCD in patients with T2DM and/or HF or CKD. However, it was not associated with lower risk of VA. Further adequately powered randomized trials are needed with more diverse participants and longer follow-ups to validate these effects of SGLT2i among T2DM and/or HF patients.

## Funding support and author disclosures

Dr Fonarow has consulted for Abbott, Amgen, AstraZeneca, Bayer, Boehringer Ingelheim, Cytokinetics, Eli Lilly, Johnson & Johnson, Medtronic, Merck, Novartis, and Pfizer. Dr Benjamin is supported in part by R01HL092577; 10.13039/100000968American Heart Association AF AHA_18SFRN34110082. Dr Michos has served on advisory boards for Amarin, AstraZeneca, Bayer, Boehringer Ingelheim, Esperion, Novartis, Novo Nordisk, and Pfizer. All other authors have reported that they have no relationships relevant to the contents of this paper to disclose.

## Supplementary data

Supplemental Table 1, and Supplemental Figures 1, 2, 3, 4, 5, 6, 7, 8, 9, 10, 11, 12, 13, 14, 15, 16, 17, 18, 19, 20, 21, 22, and 23
