# Supplementary material for: Sodium-Glucose Cotransporter-2 Inhibitors and Arrhythmias: A Meta-Analysis of 38 Randomized Controlled Trials
Source: JACC Adv. 2025 Feb 22;4(3):101615. doi: 10.1016/j.jacadv.2025.101615 (PMC11904486; doi:10.1016/j.jacadv.2025.101615)
Supplement: Supplemental Table 1, and Supplemental Figures 1, 2, 3, 4, 5, 6, 7, 8, 9, 10, 11, 12, 13, 14, 15, 16, 17, 18, 19, 20, 21, 22, and 23 [file mmc1.docx]

**Supplemental Table 1:** Search strategy

| **Electronic database** | **Search strategy** |
| --- | --- |
| Pubmed | (((((((((((((heart failure[MeSH Terms]) OR (HFpEF[Other Term])) OR (HFrEF[Other Term])) AND (diabetes mellitus[MeSH Terms])) OR (T2DM[Other Term])) AND (chronic kidney disease[Other Term])) AND (SGLT2 inhibitors[Other Term])) OR (Sodium-Glucose Cotransporter-2 Inhibitors[Other Term]) OR (arrhythmia[MeSH Terms])) OR (atrial fibrillation[MeSH Terms])) OR (ventricular fibrillation[MeSH Terms])) OR (atrial flutter[MeSH Terms])) OR (ventricular flutter[MeSH Terms])) AND (sudden cardiac death[MeSH Terms])) AND (cardiac arrest[MeSH Terms])) AND (controlled clinical trials, randomized[MeSH Terms]) |

A) Risk of Bias Summary


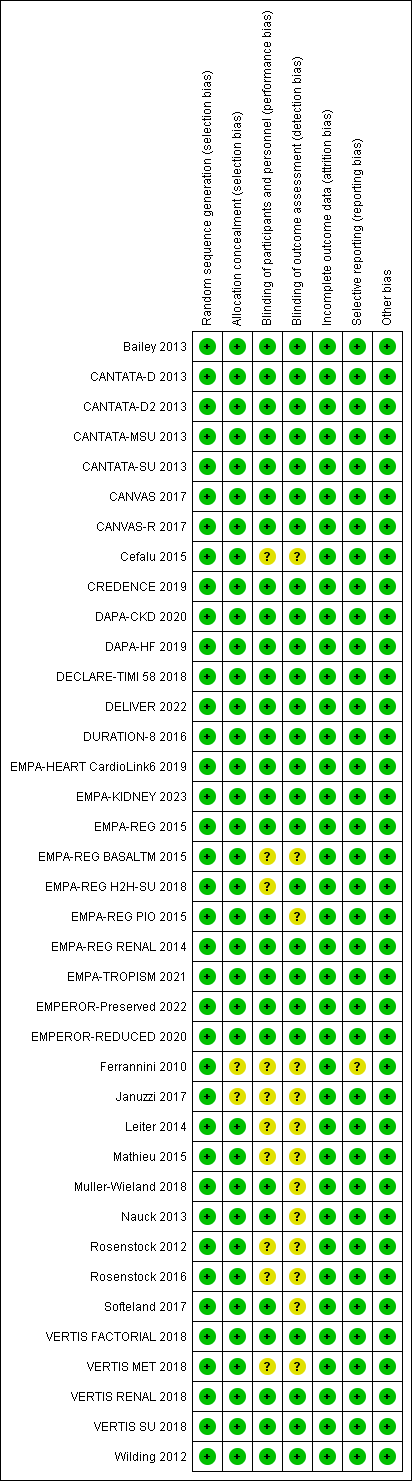


B) Risk of Bias Graph


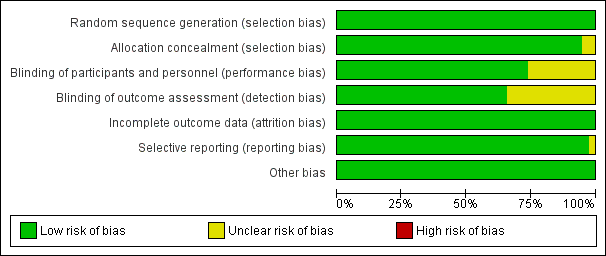


**Supplemental Figure 1** Cochrane risk of bias including A) Risk of Bias summary, B) Risk of Bias graph

A) Atrial fibrillation


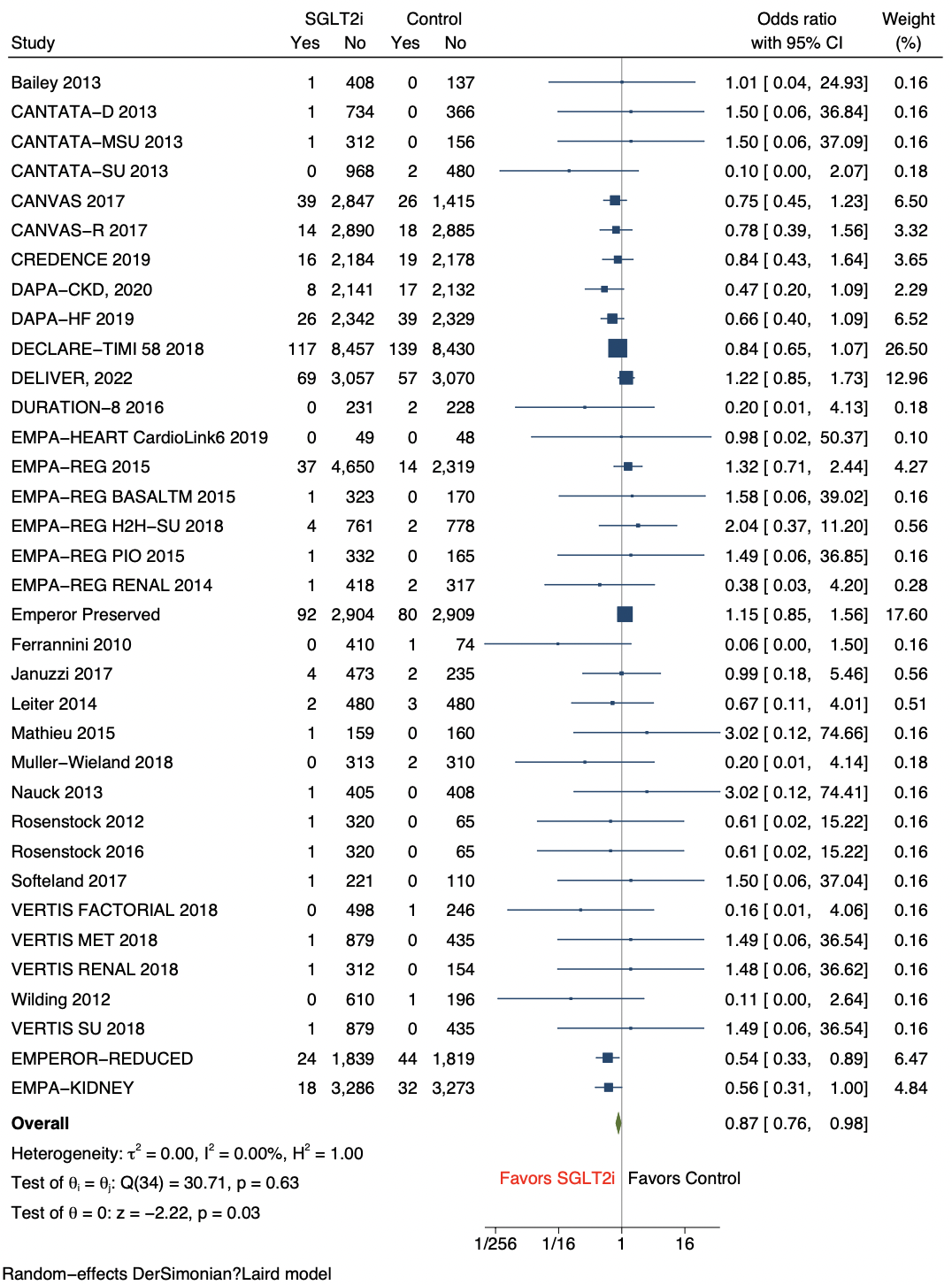


B) Atrial flutter

**
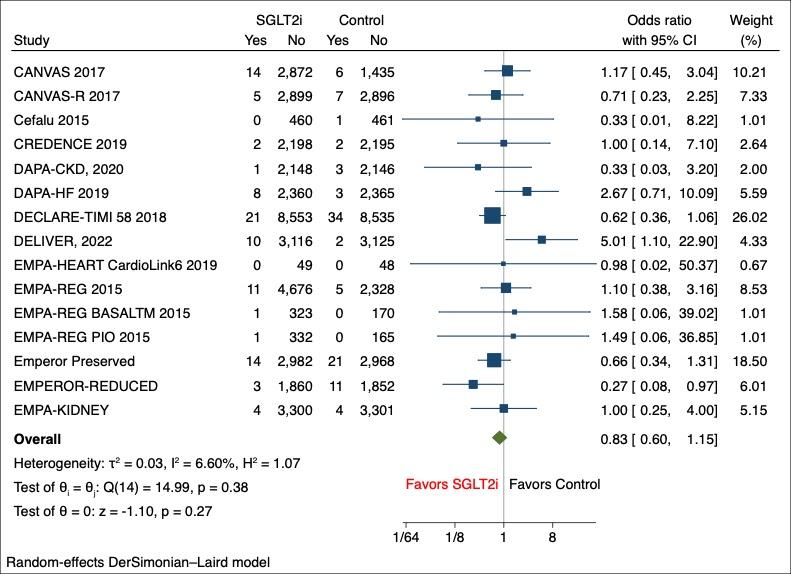
**

**Supplemental Figure 2** Forest plots of subanalysis on type of Atrial arrhythmia including A) Atrial fibrillation, B) Atrial flutter

A) Ventricular fibrillation


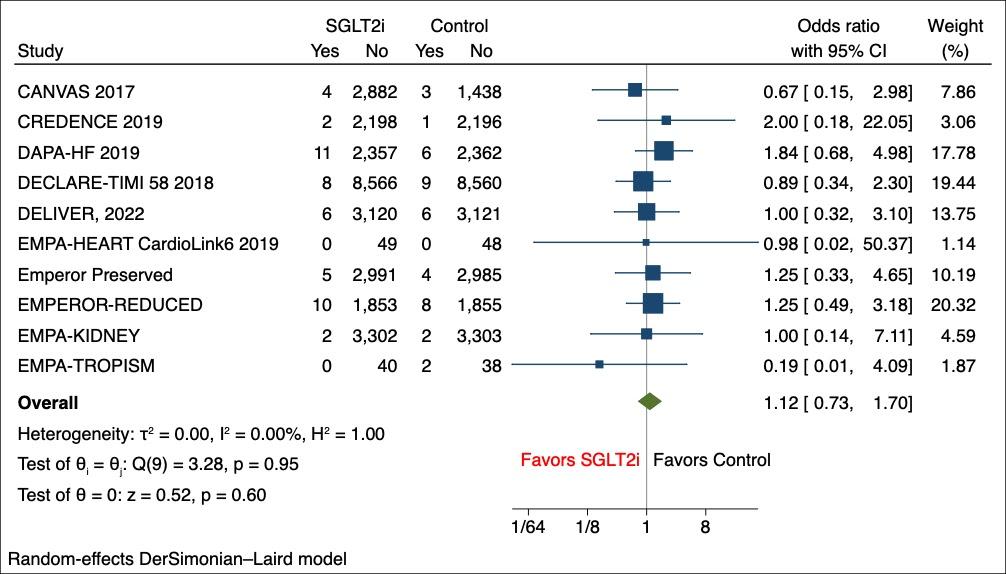


B) Ventricular flutter


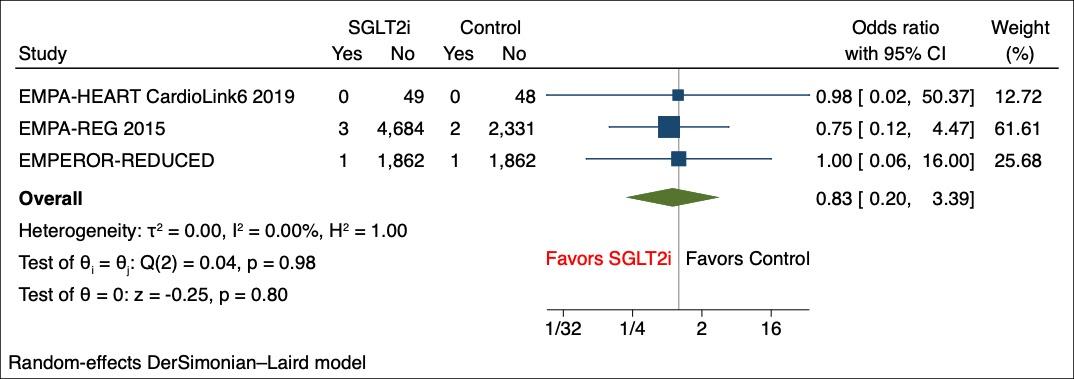


C) Ventricular tachycardia

**
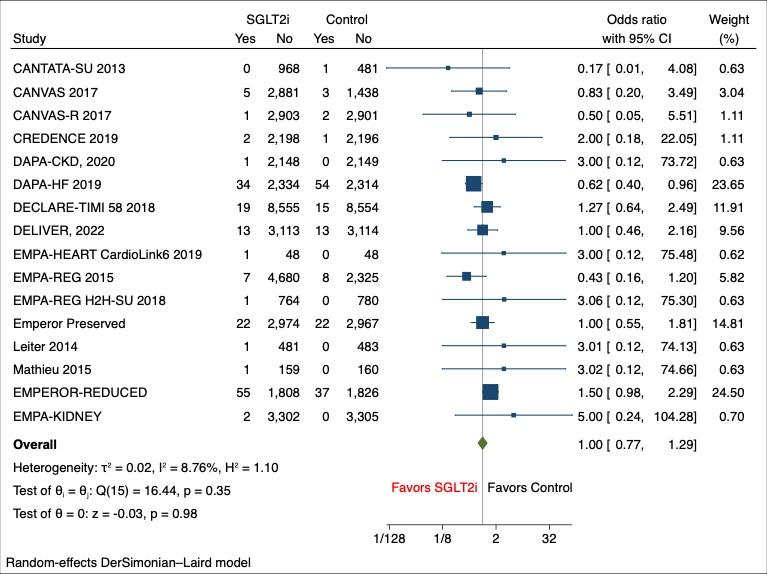
**

**Supplemental Figure 3** Forest plots of subanalysis on type of Ventricular arrhythmia including A) Ventricular fibrillation, B) Ventricular flutter, C) Ventricular tachycardia

**
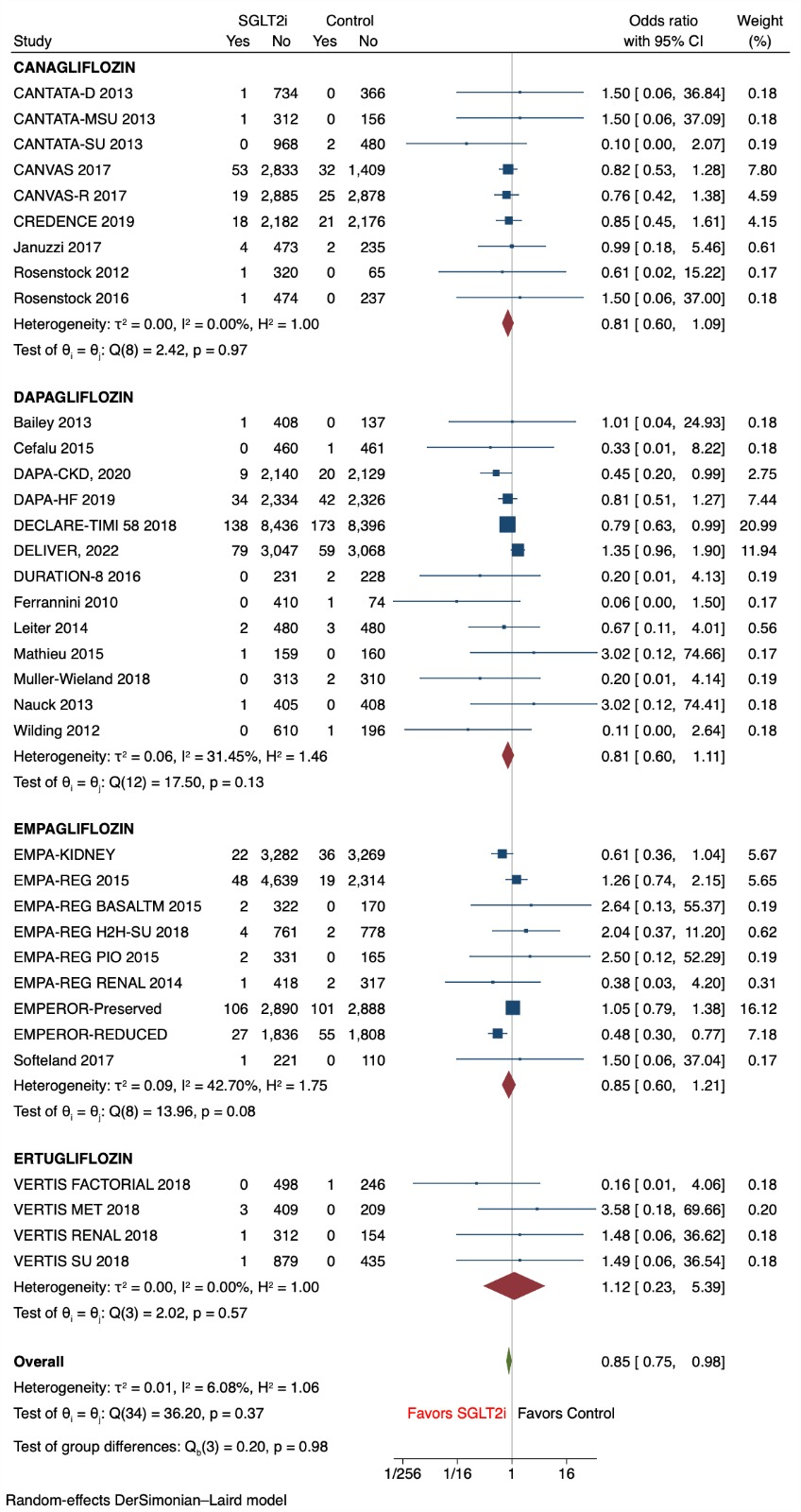
**

**Supplemental Figure 4** Subgroup analyses on Atrial arrhythmia based on types of SGLT2i.

**
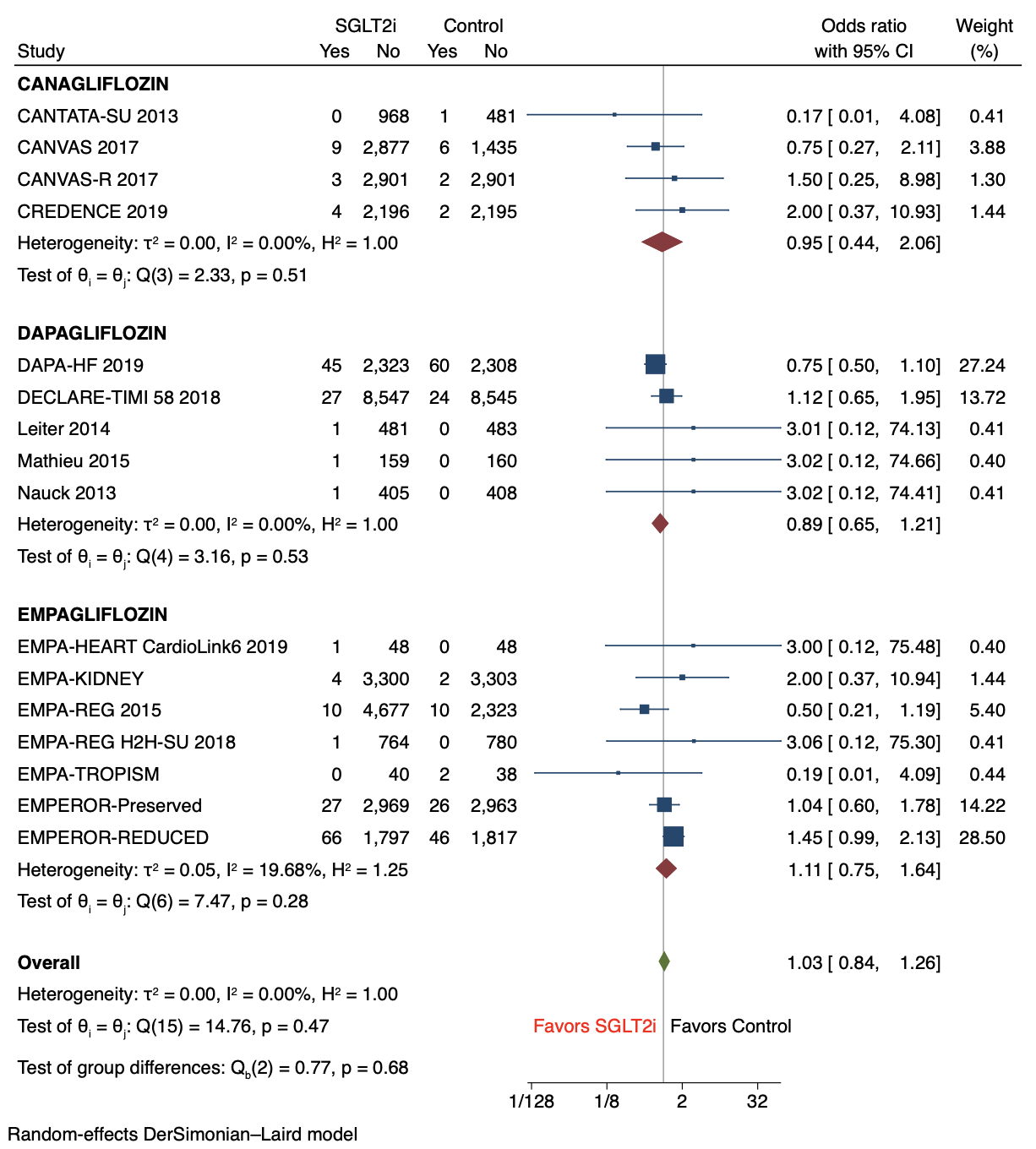
**

**Supplemental Figure 5** Subgroup analyses on Ventricular arrhythmia based on type of SGLT2i

**
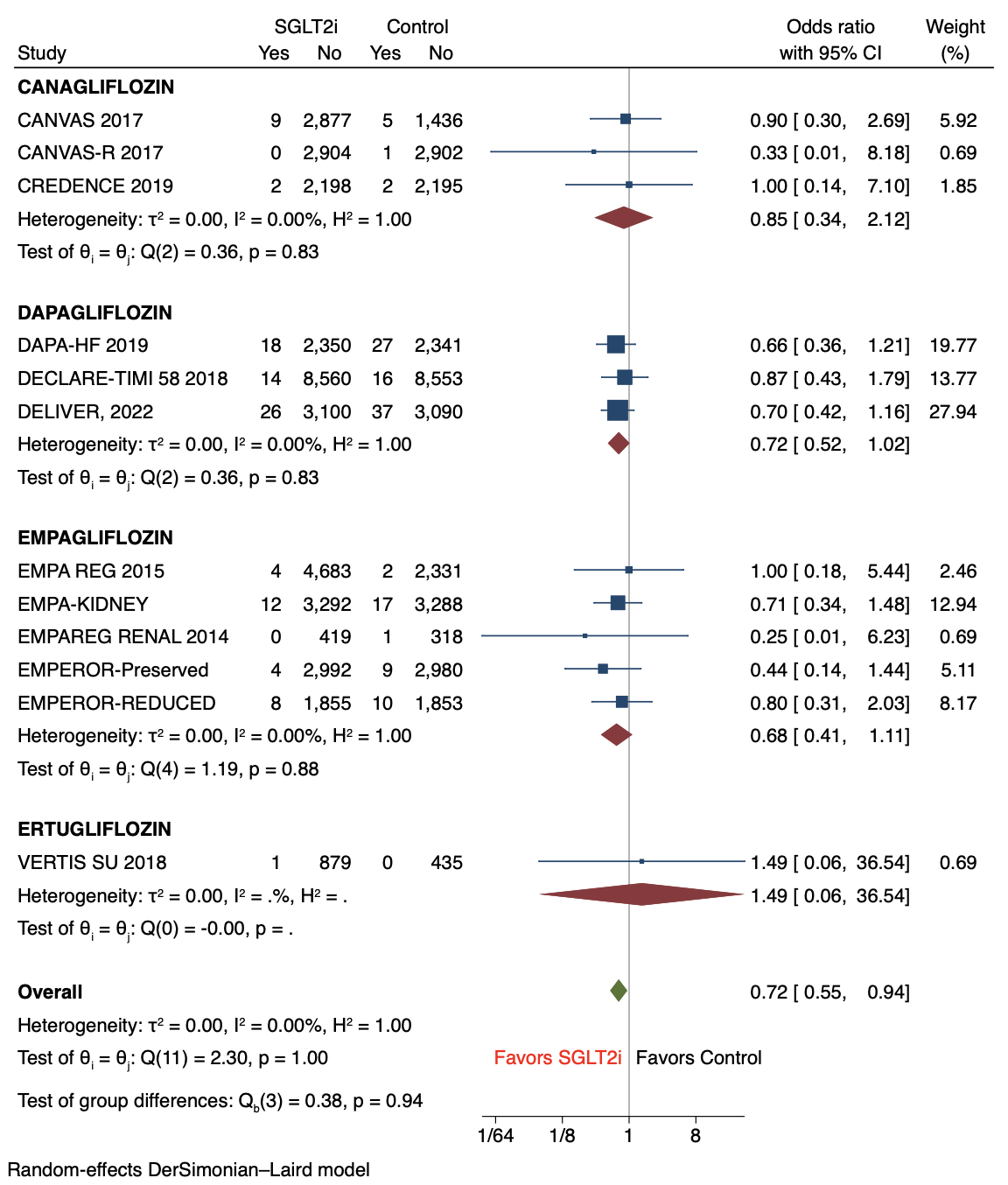
**

**Supplemental Figure 6** Subgroup analyses on SCD based on type of SGLT2i

**
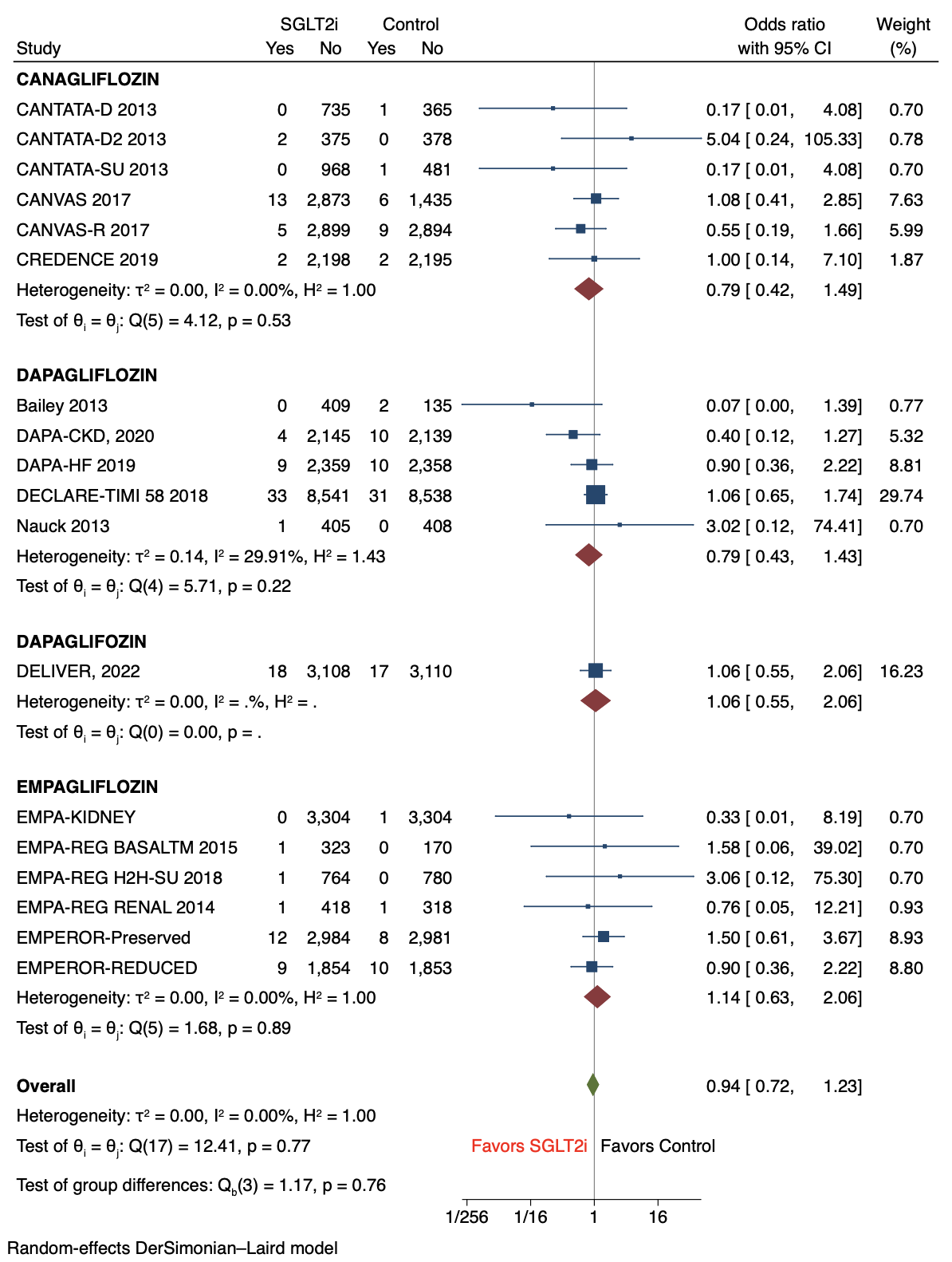
**

**Supplemental Figure 7** Subgroup analyses on Cardiac arrest based on type of SGLT2i

**
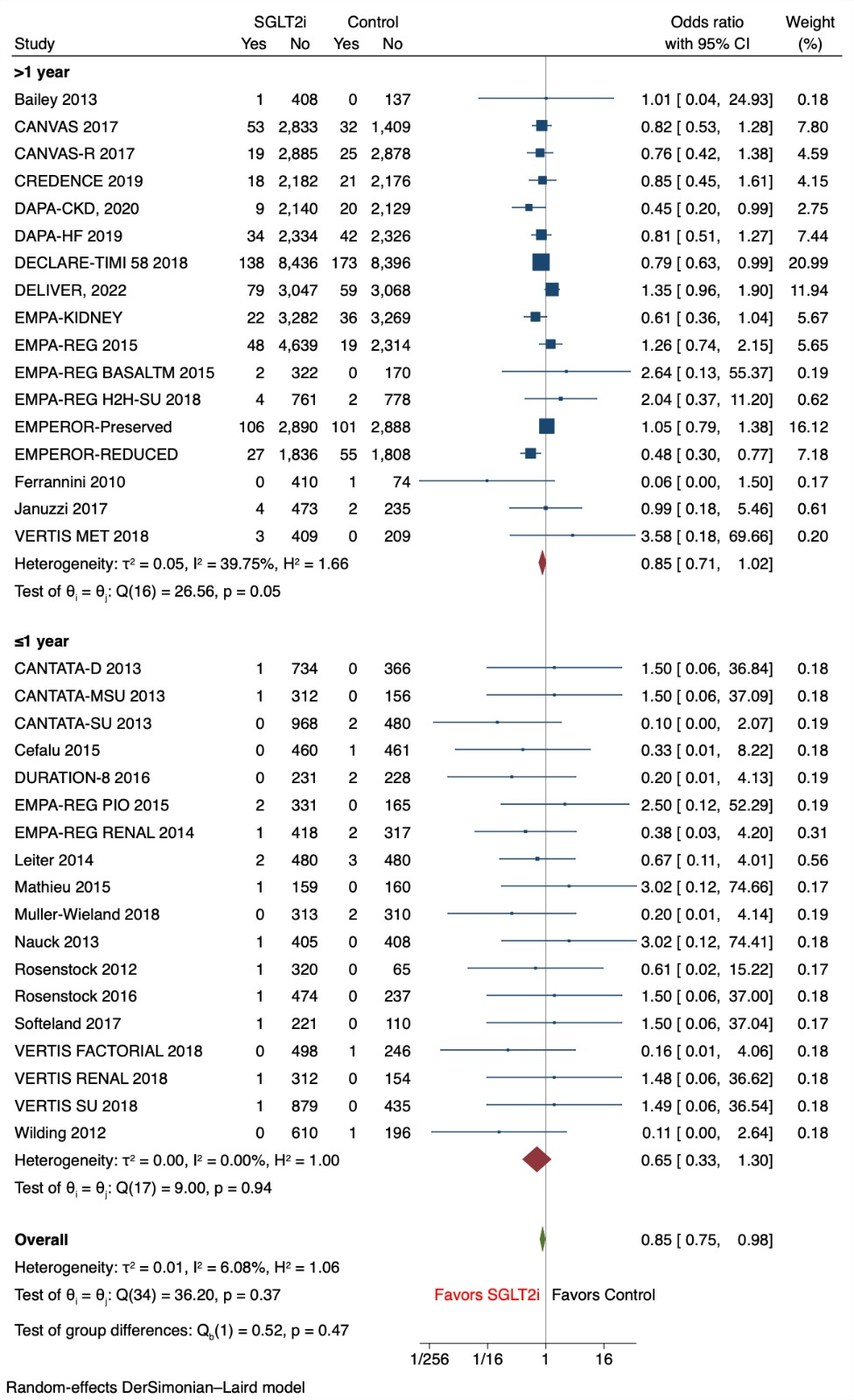
**

**Supplemental Figure 8** Subgroup analyses on Atrial arrhythmia based on follow-up period

**
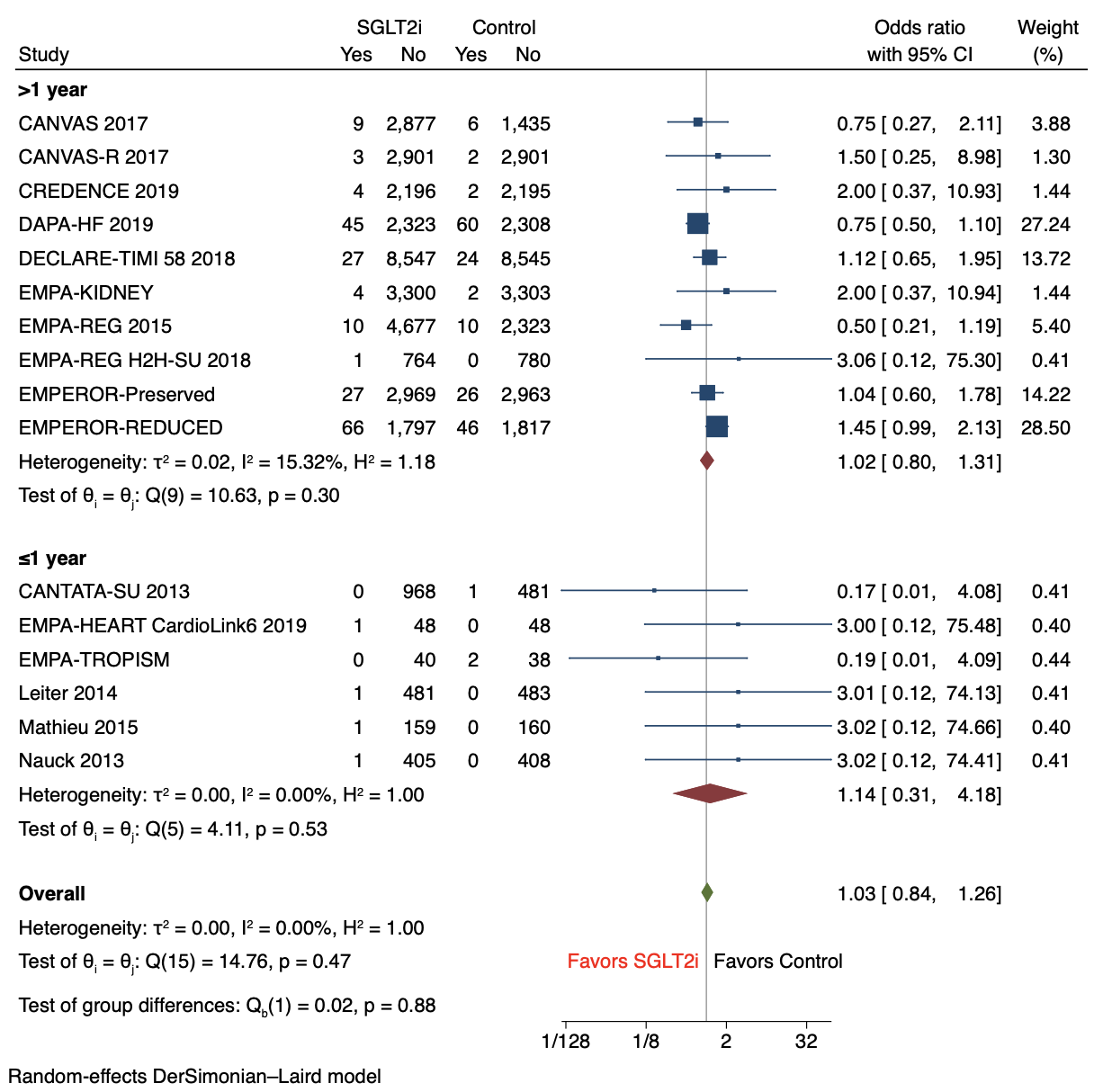
**

**Supplemental Figure 9** Subgroup analyses on Ventricular arrhythmia based on follow-up period

**
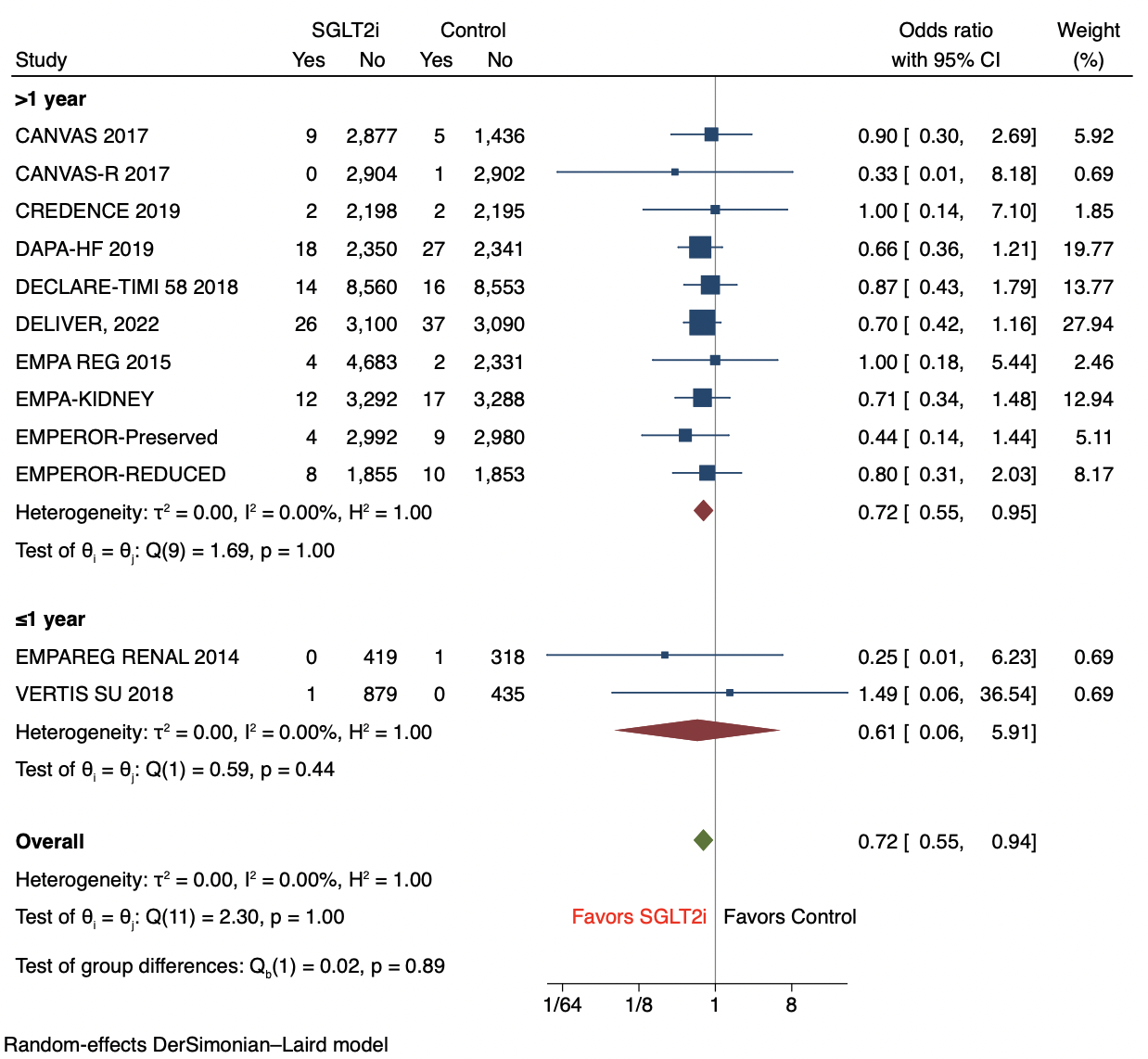
**

**Supplemental Figure 10** Subgroup analyses on SCD based on follow-up period

**
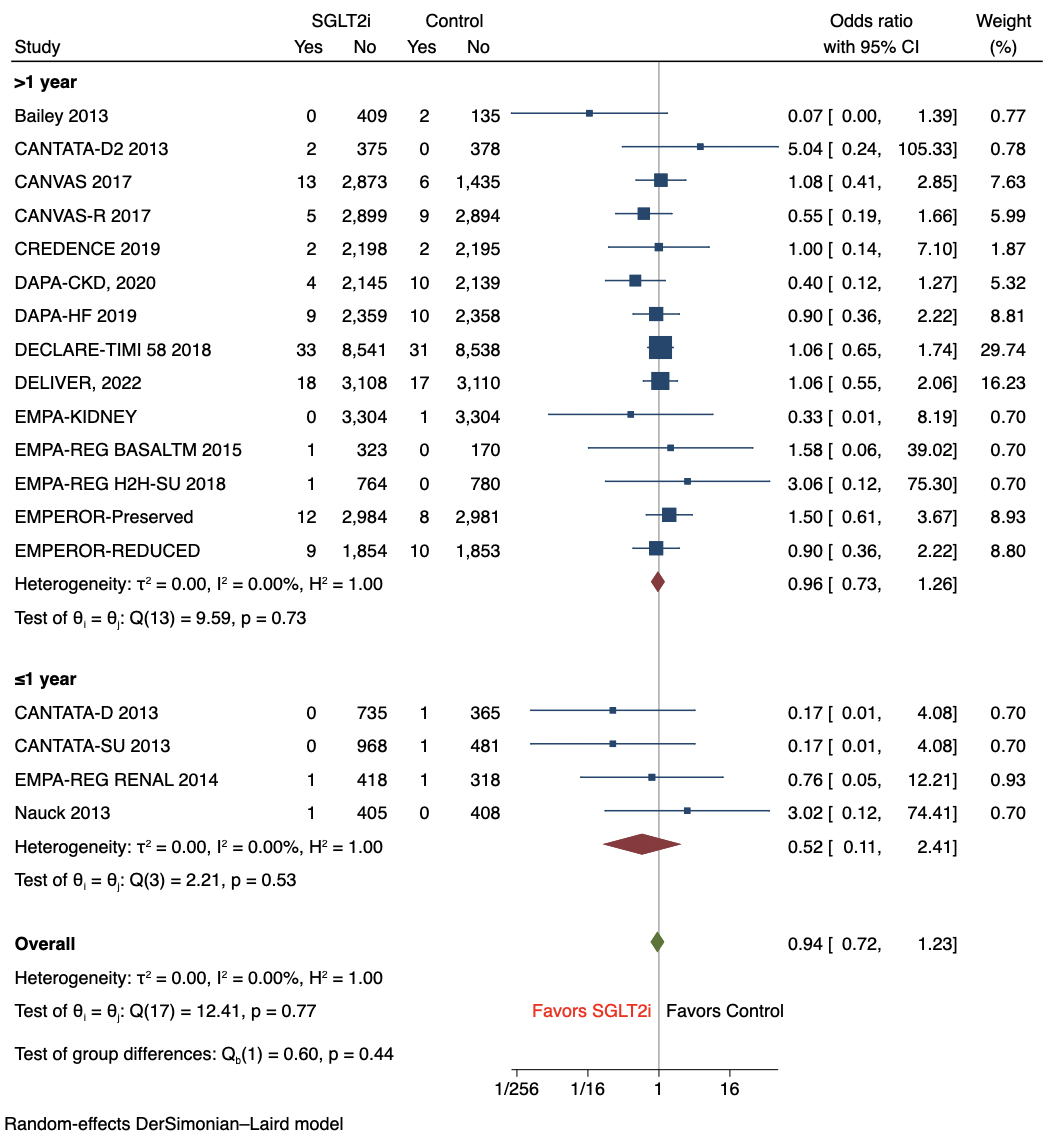
**

**Supplemental Figure 11** Subgroup analyses on Cardiac arrest based on follow-up period

**
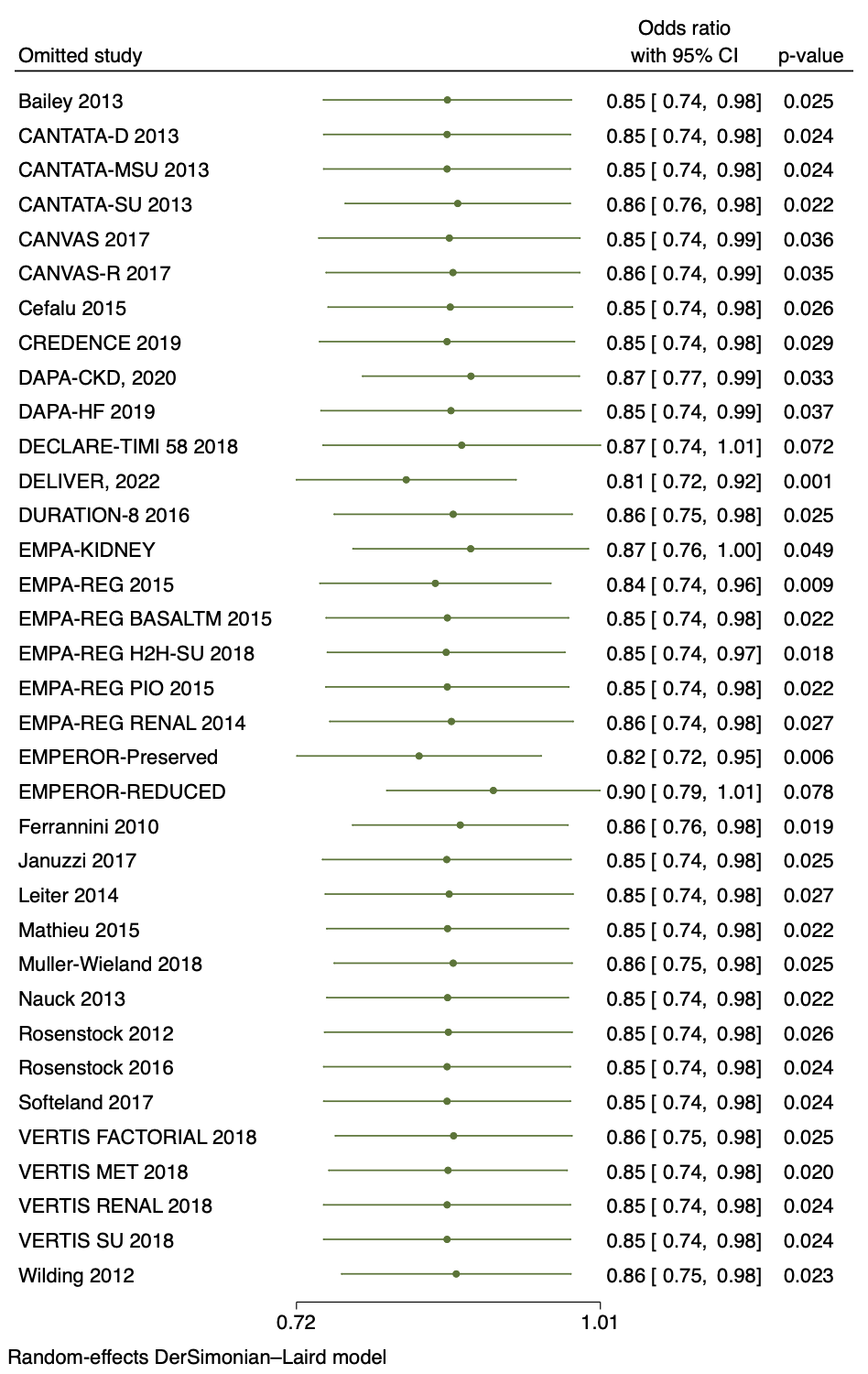
**

**Supplemental Figure 12** Leave-one-out analysis for Atrial arrhythmia


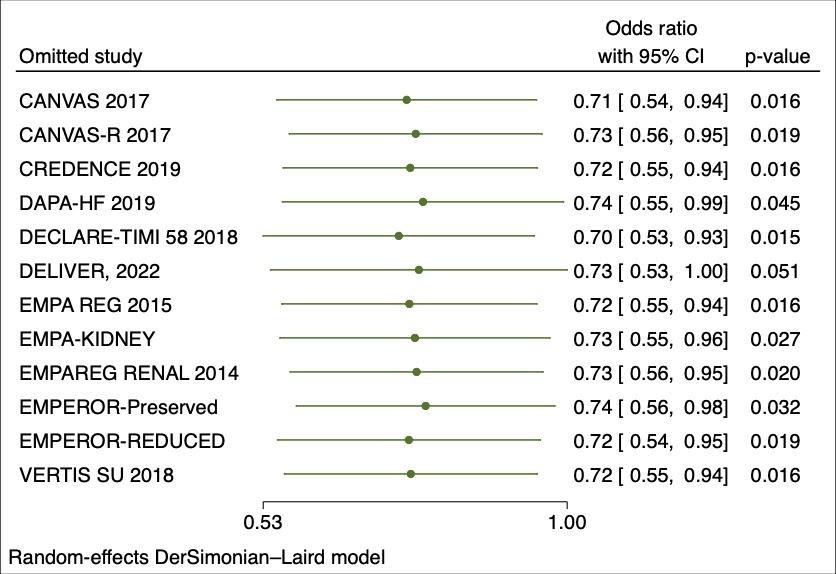


**Supplemental Figure 13** Leave-one-out analysis for SCD

**
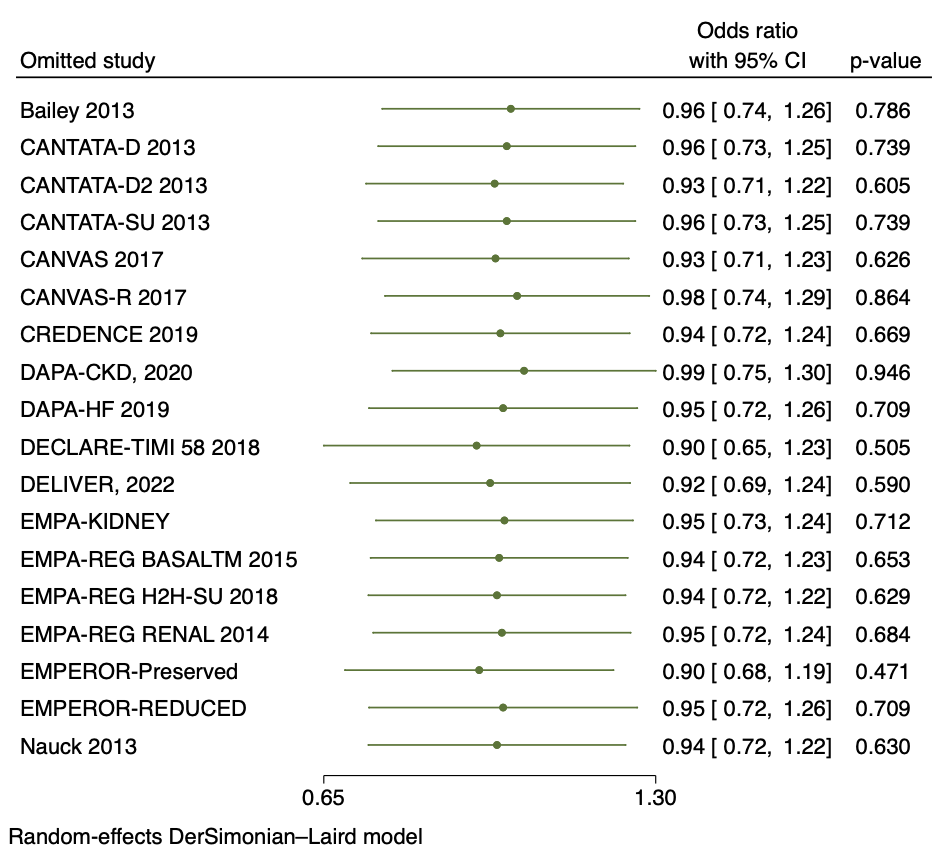
**

**Supplemental Figure 14** Leave-one-out analysis for Cardiac arrest

**
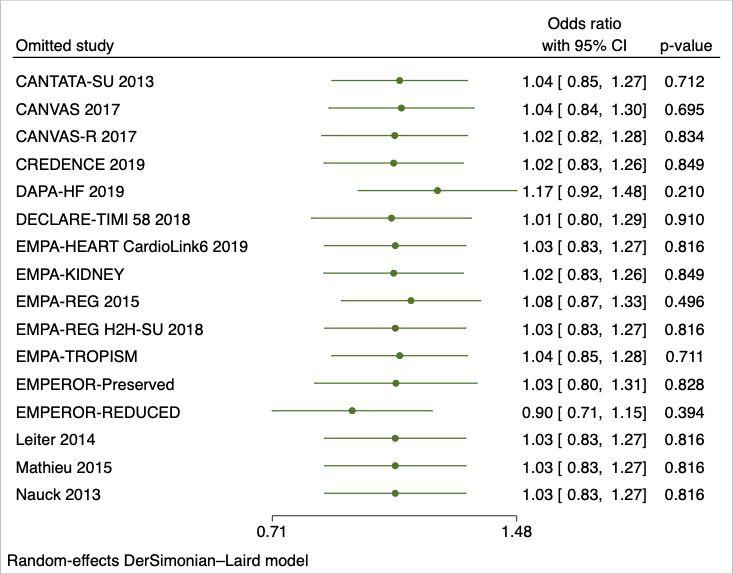
**

**Supplemental Figure 15** Leave-one-out analysis for Ventricular arrhythmia

**
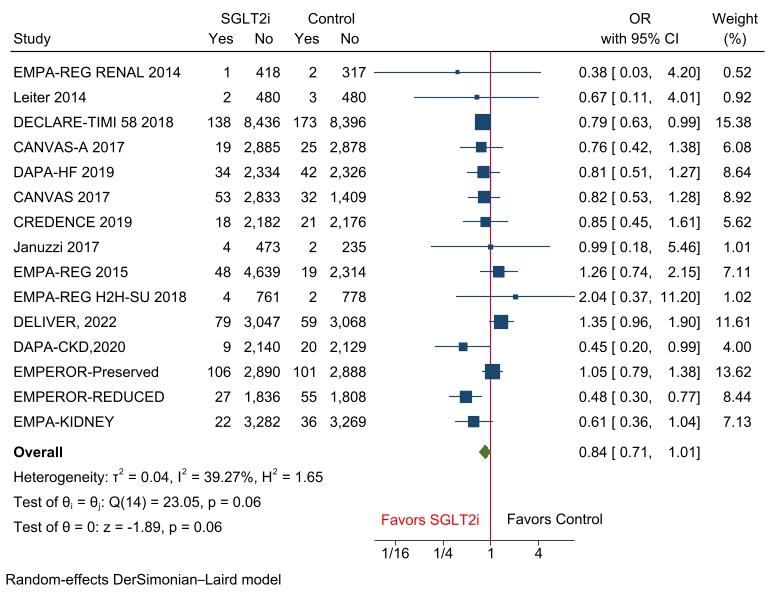
**

**Supplemental Figure 16.** Pooled analysis after removal of zero events for Atrial arrhythmia.


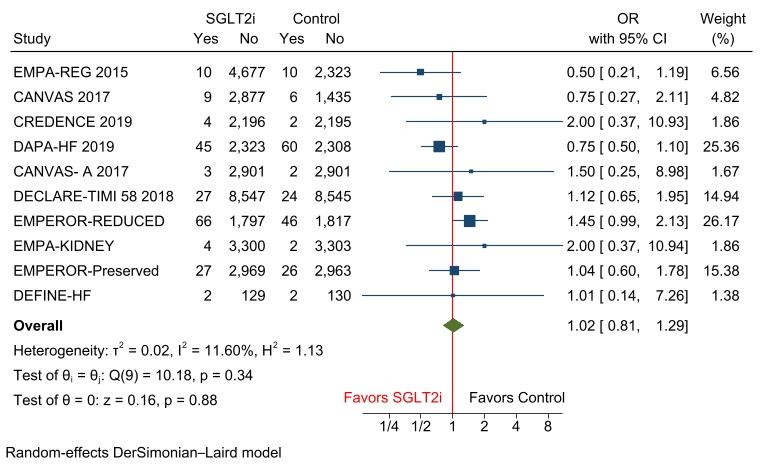


**Supplemental Figure 17.** Pooled analysis after removal of zero events for Ventricular arrhythmia.


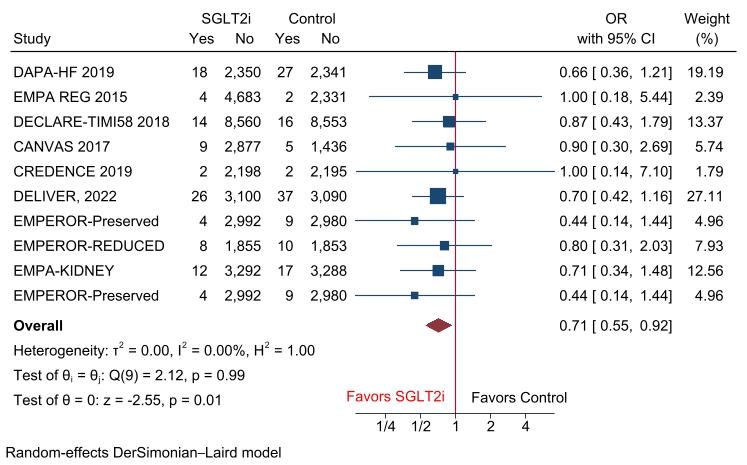


**Supplemental Figure 18.** Pooled analysis after removal of zero events for Sudden Cardiac Death.


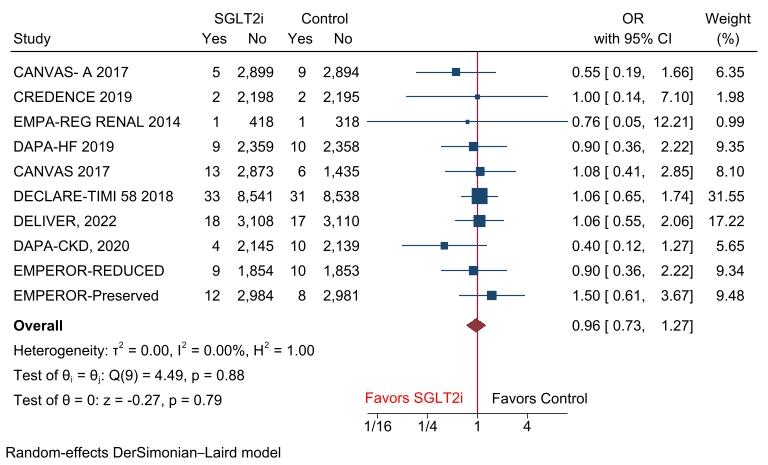


**Supplemental Figure 19.** Pooled analysis after removal of zero events for Cardiac Arrest.

**
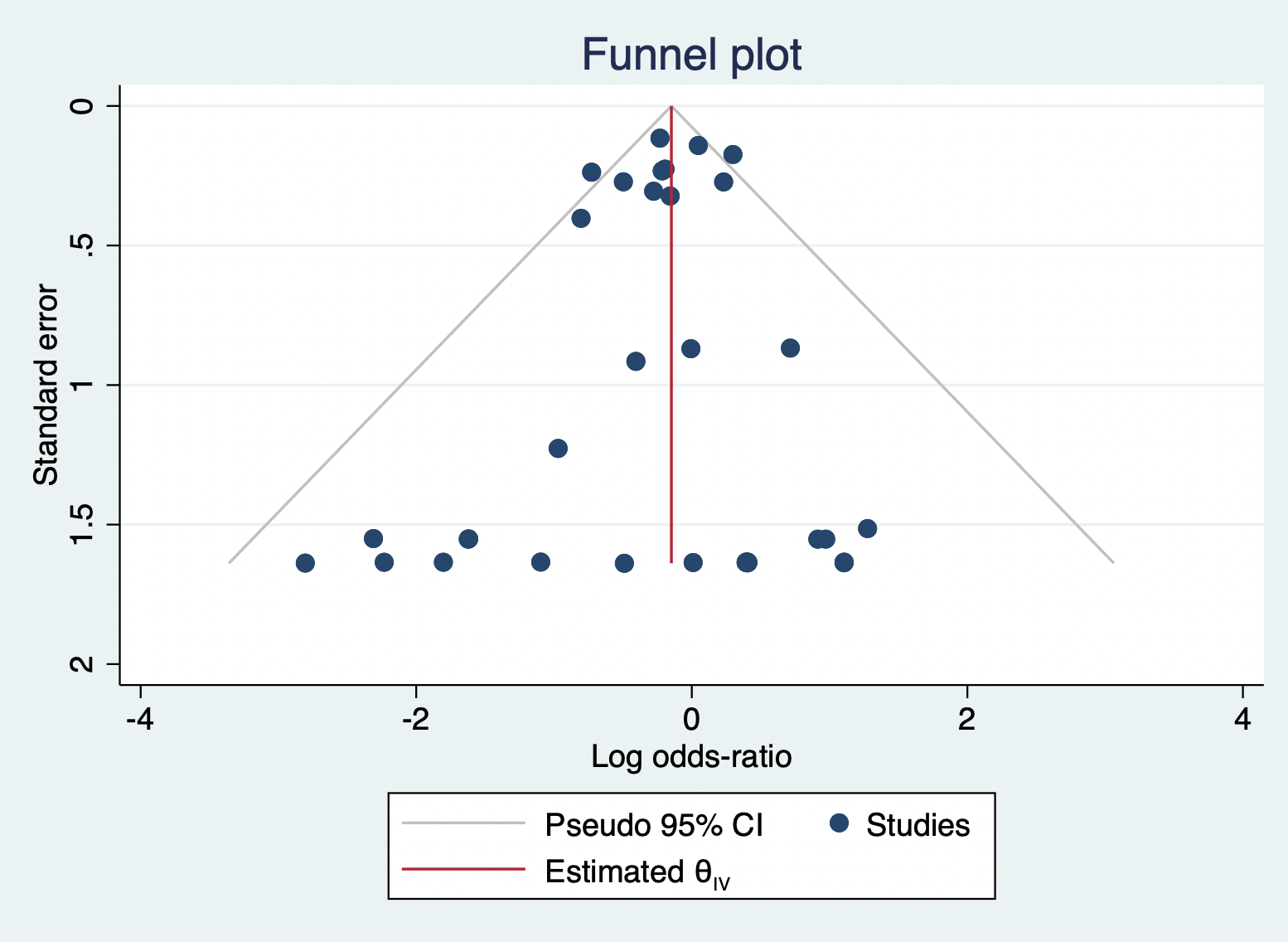
**

**Supplemental Figure 20.** Funnel plot for Atrial arrhythmia

**
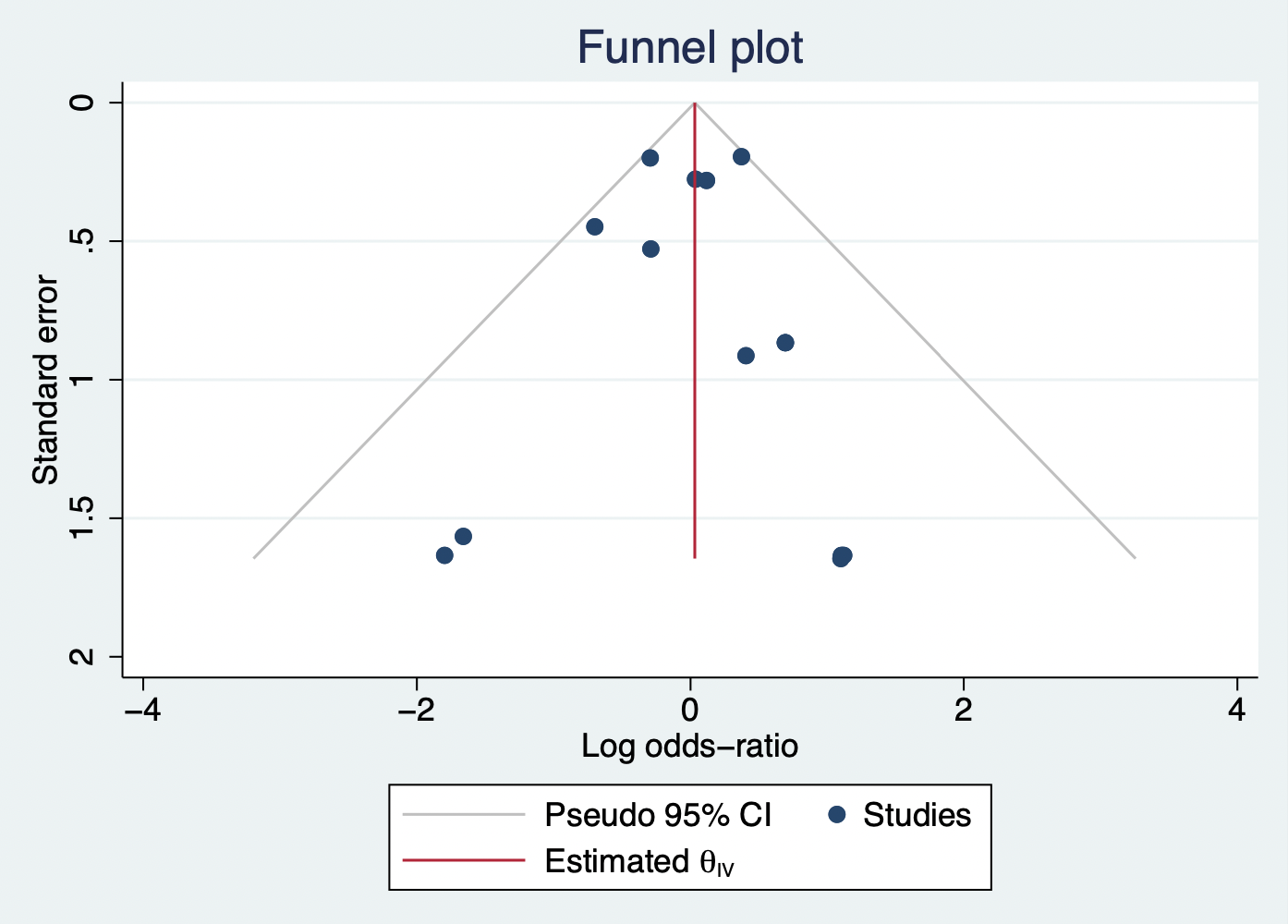
**

**Supplemental Figure 21.** Funnel plot for Ventricular arrhythmia


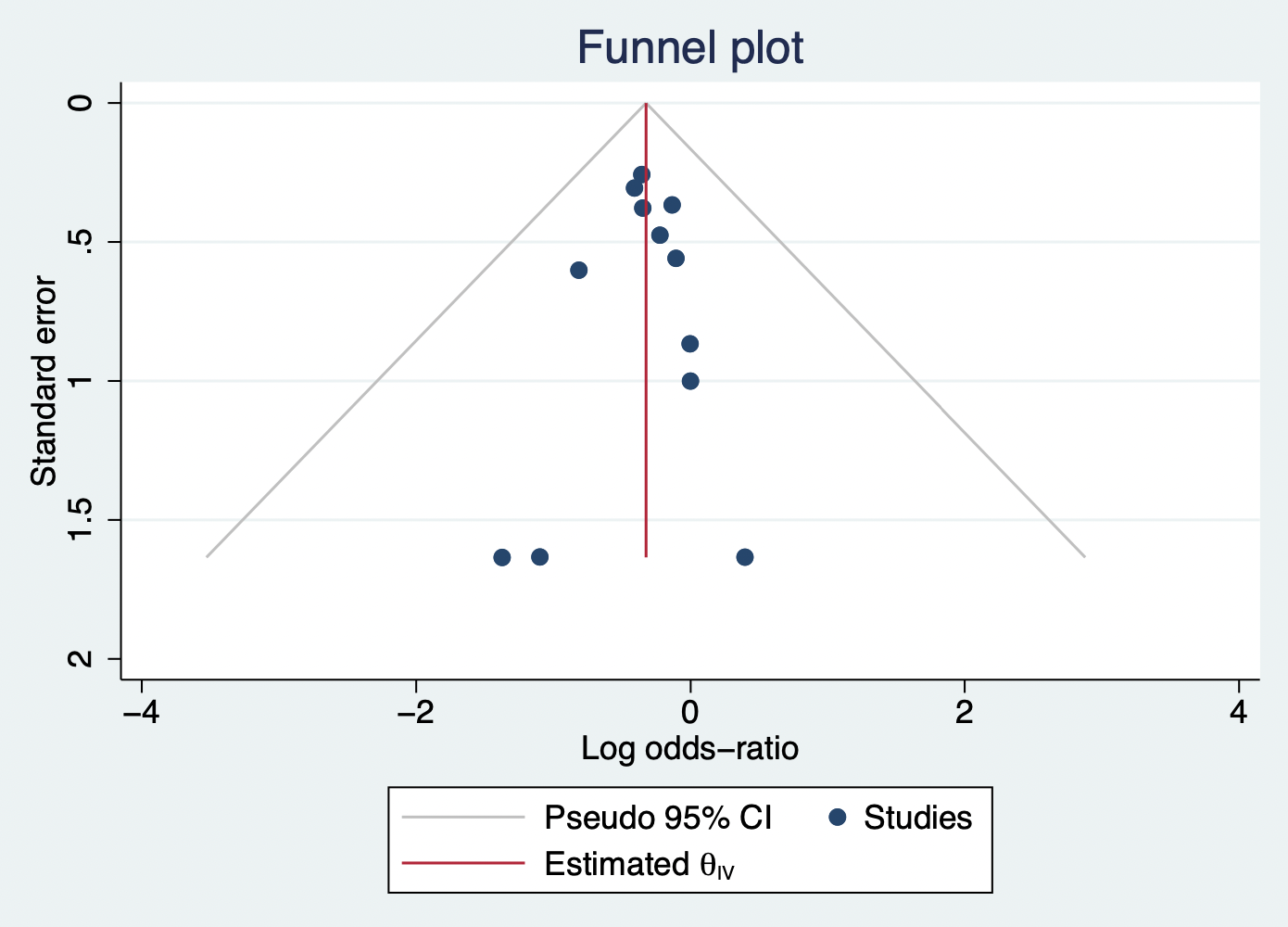


**Supplemental Figure 22.** Funnel plot for SCD


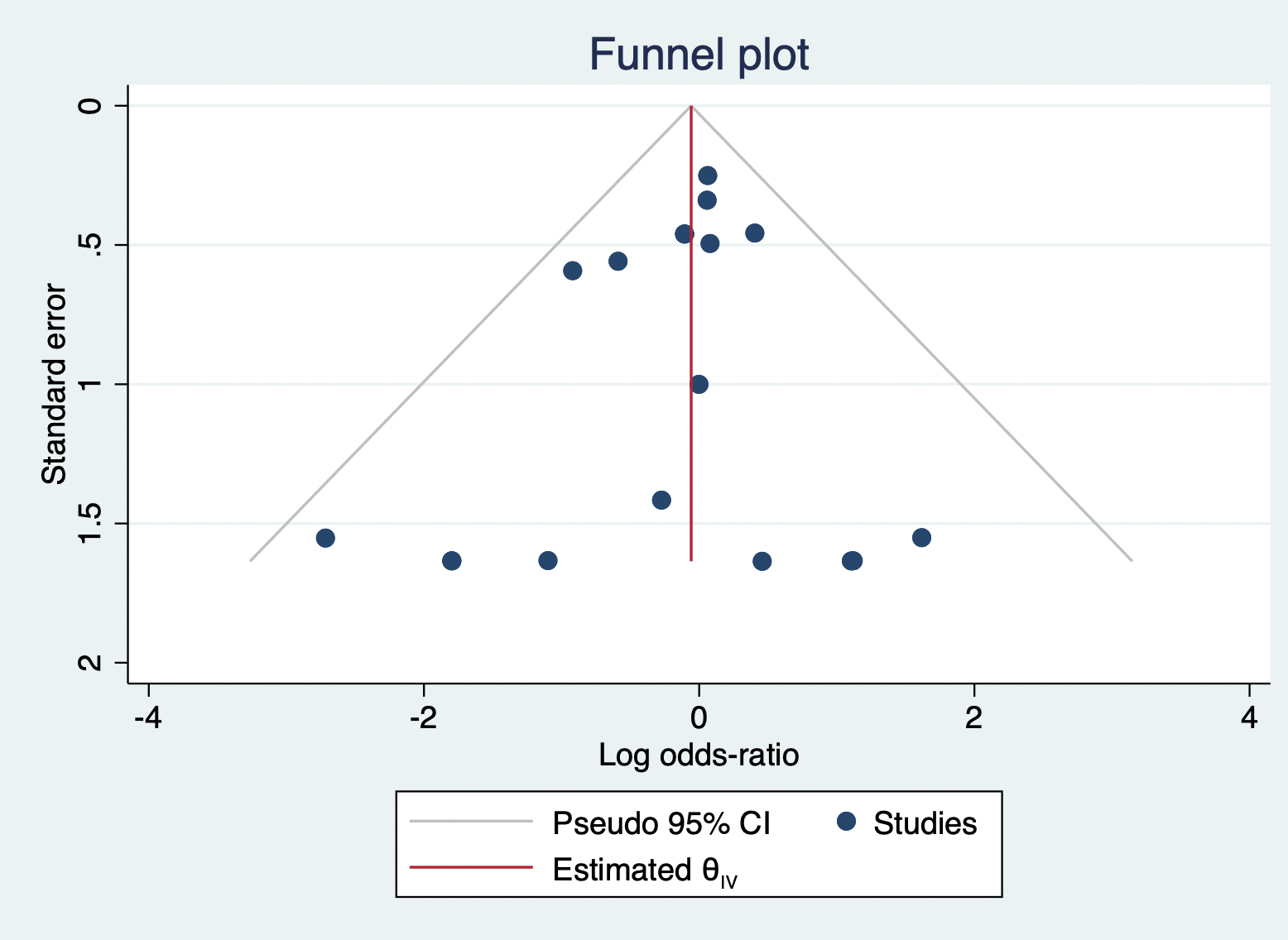


**Supplemental Figure 23.** Funnel plot for Cardiac arrest
